# Supplementary material for: Bilayer-Mediated Structural Transitions Control Mechanosensitivity of the TREK-2 K2P Channel
Source: Structure. 2017 May 2;25(5):708–718.e2. doi: 10.1016/j.str.2017.03.006 (PMC5415359; doi:10.1016/j.str.2017.03.006)
Supplement: Document S1. Figures S1–S5 [file mmc1.pdf]

Structure, Volume *25*

Supplemental Information

Bilayer-Mediated Structural Transitions Control Mechanosensitivity of the TREK-2 K2P Channel

Prafulla Aryal, Viwan Jarerattanachat, Michael V. Clausen, Marcus Schewe, Conor McClenaghan, Liam Argent, Linus J. Conrad, Yin Y. Dong, Ashley C.W. Pike, Elisabeth P. Carpenter, Thomas Baukrowitz, Mark S.P. Sansom, and Stephen J. Tucker

Supplementary Information

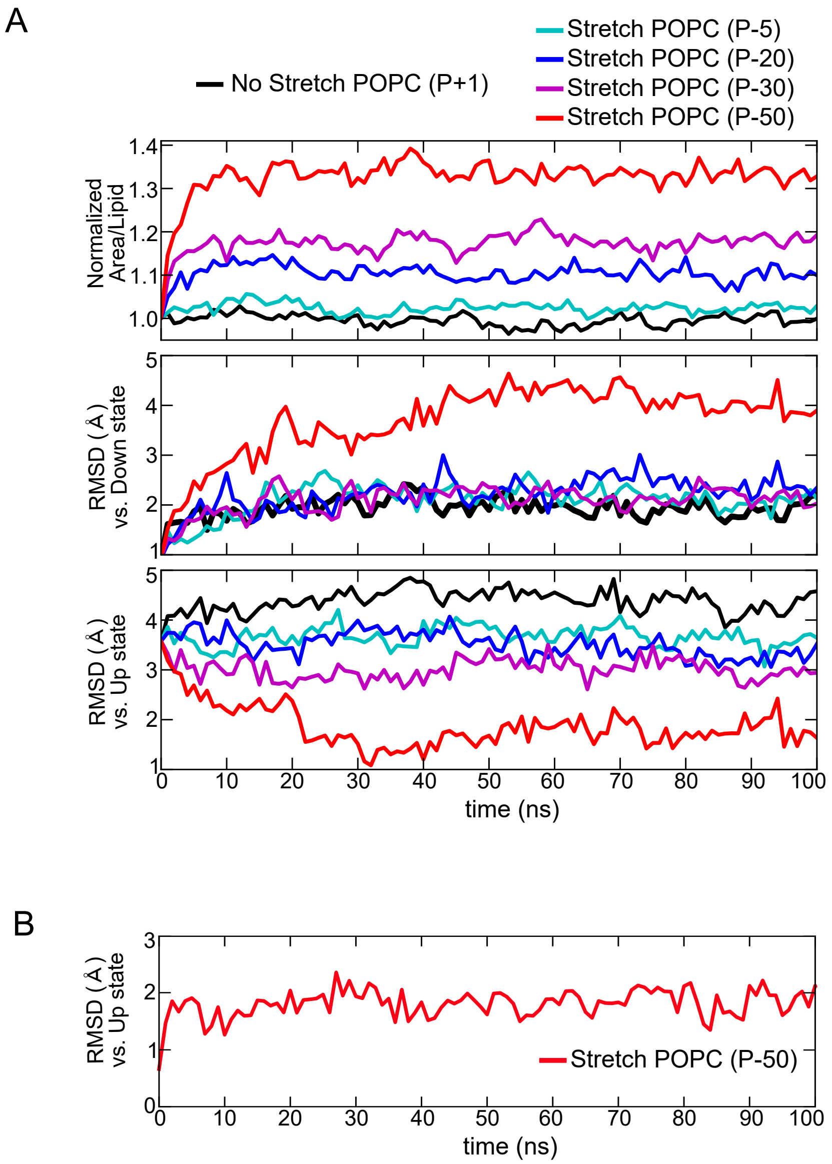

Figure S1. Related to Figure 1.

Development of stretch protocol. (**A**) Top: Increase in bilayer surface area per lipid in response to application of increasing levels of membrane stretch. Bilayer surface tensions have been estimated by using $\gamma = Z(P_{\perp} - P_{\parallel})$, where Z is average box size in bilayer normal direction, $P_{\perp}$ and $P_{\parallel}$ is pressure in bilayer normal and bilayer plane, respectively. The estimated average bilayer tensions correlating with the pressure

protocols described in this figure (P-5, P-20, P-30, and P-50 bar) are: 7.6, 24.6, 34.3, and 50.3 mN m^{-1}, respectively. Middle: Cα RMSD for M1-M4 of TREK-2 against the Down state crystal structure during 200 ns of simulation for different levels of membrane stretch in a POPC bilayer. At P-50 (-50 bar) there is rapid movement towards the Up state within 50 ns, but not in the absence of stretch (black). Bottom: Similar analysis of RMSD against the Up state crystal structure showing movement towards the Up state at different membrane tensions. (**B**) Simulation of the Up state structure (4BW5) as the starting conformation shows that this conformation is extremely stable in response to the P-50 stretch protocol and exhibits little or no movement of the TM-helices (RMSD 1-2 Å).

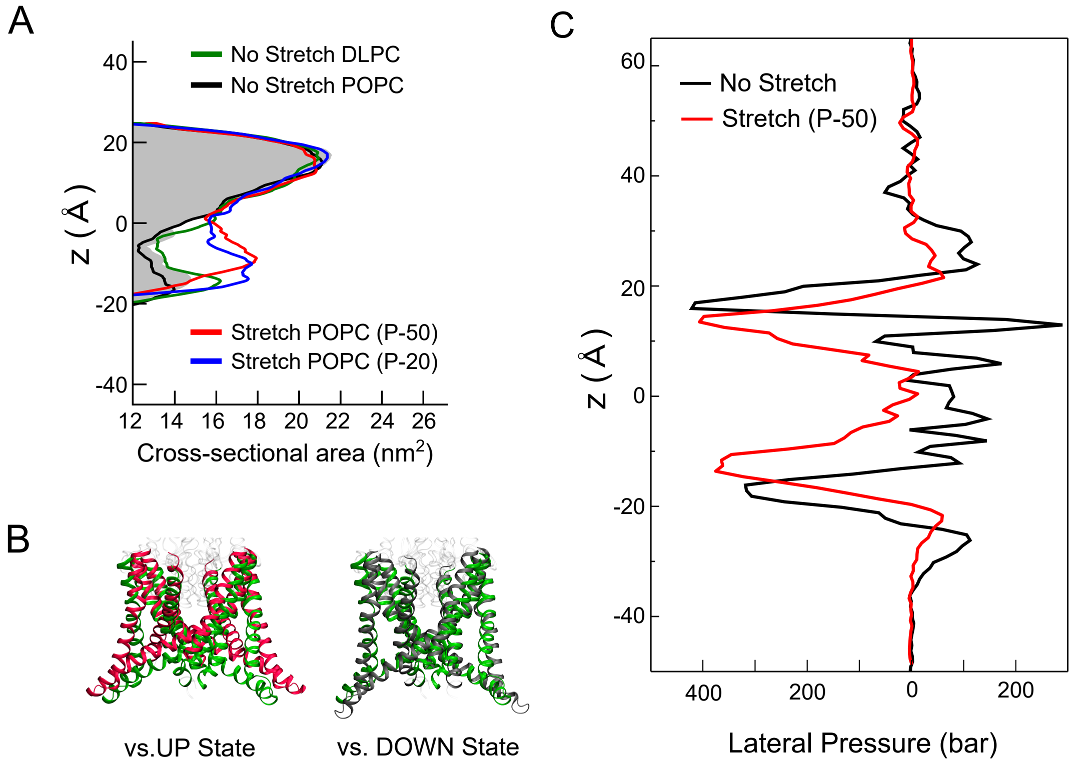

Figure S2. Related to Figure 2.

Effect of changes in the pressure profile and bilayer thickness. (**A**) Change in cross-sectional area of TREK-2 Down state during simulation in the different bilayers used in this study. Stretch at -50 bar (red) and also -20 bar (blue) produce similar increases in cross-sectional area but over different timescales. By marked contrast, the thinner DLPC bilayer (i.e. hydrophobic mismatch) is not sufficient to drive movement towards the Up state (green). Grey shaded area represents the Down state crystal structure (4XDJ). (**B**) This lack of movement is also illustrated by comparison of the TREK-2 structure after simulation in a DLPC bilayer (green) with the Up (red) and Down state (dark grey) crystal structures. (**C**) Pressure profiles calculated for stretched (red) and unstretched (black) POPC bilayers with TREK-2 protein embedded within the bilayer. These profiles are more complex but membrane stretch still reduces the positive pressures within the core of the bilayer and changes the tension at the interfacial regions.

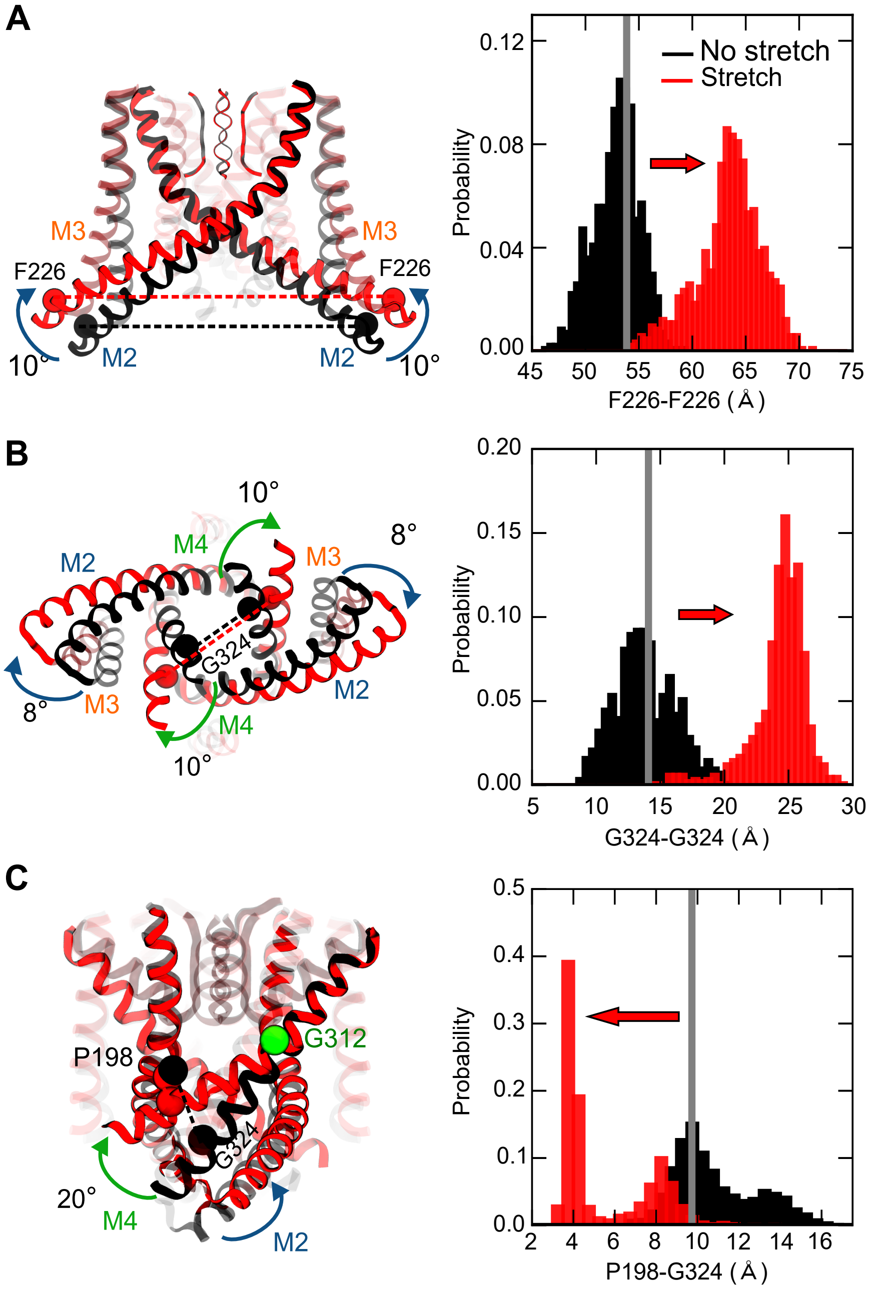

Figure S3. Related to Figure 3.

Stretch induced changes in the structure of TREK-2. (**A**) Left: Stretch increases the tilt angle of the bow-like M2 helices by 10° and also increases the distance between them (measured between F226 residues). Right: Distribution of distances between F226

during stretched (red) and unstretched simulations (black) showing >10 Å increase upon stretched. Vertical grey line indicates distance in the Down state crystal structure. Histograms represent the distribution of distances between the specified residues (sampled every 1 ns) during the stretched and unstretched simulations. (**B**) Rotated view up into channel pore revealing additional 8° twist in M2 (blue arrow) and 10° twist in M4 (green arrow). Stretch increases the distance between G324 residues on M4 as also shown in the distributions. (**C**) Side view showing the 20° kink in M4 upon membrane stretch. M4 kinks at the conserved glycine hinge (G312; green dot) and subsequent movement towards M2 closes and seals the fenestration. Distance distributions show closure of fenestration upon membrane stretch (measured between G324 on M4 and P198 on M2 of the adjacent chain).

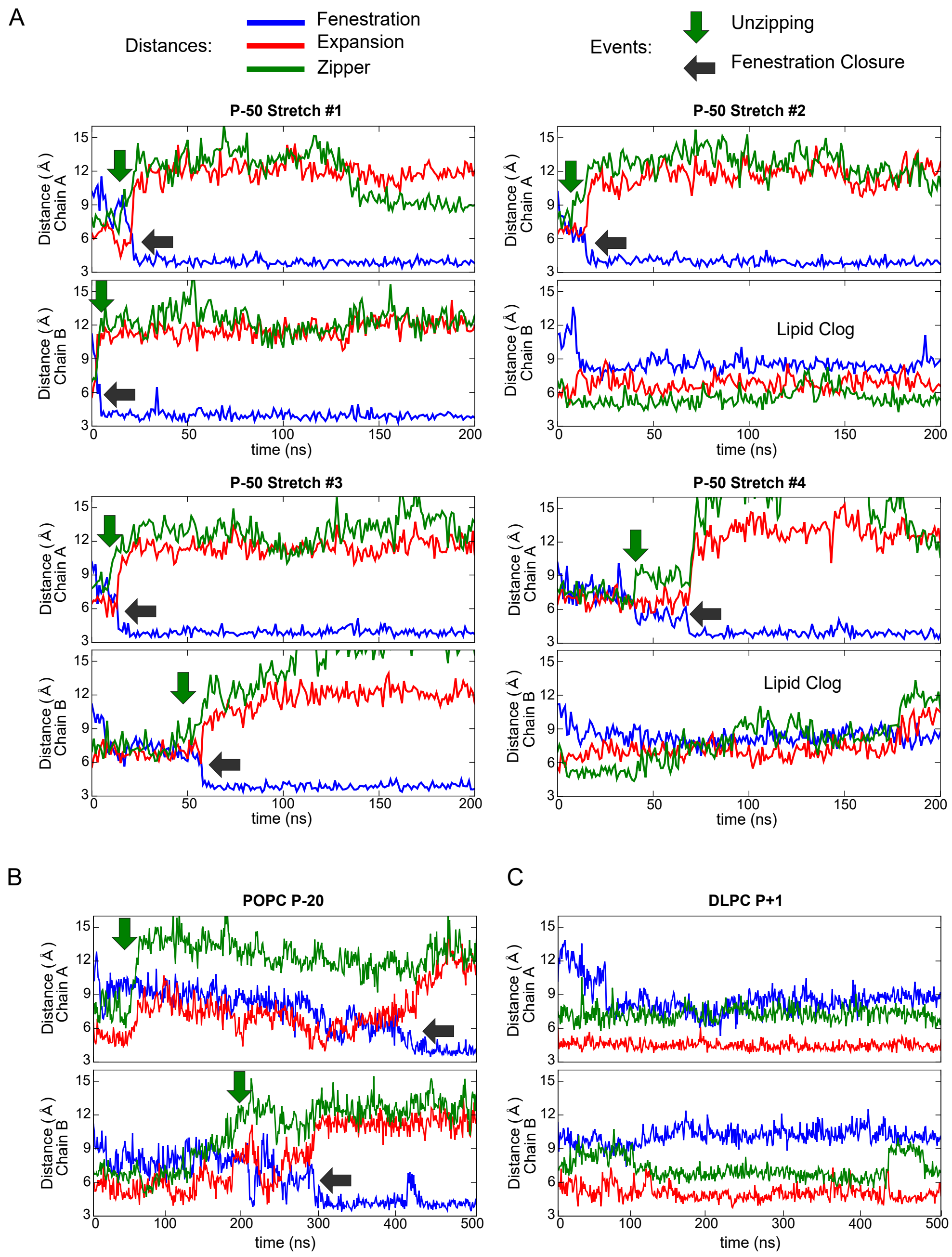

Figure S4. Related to Figure 3.

Sequential and stepwise rearrangement of the TM helices in response to membrane stretch. (**A**) Similar to the data shown in Figure 3B, changes in the fenestration (blue), expansion (red) and zipper (green) distances over time for the 4 stretch simulations at -50 bar in a POPC bilayer. Profiles for both chains A and B within each TREK-2 channel are shown. Green arrows indicate the ‘unzipping’ of the

interactions between the lower sections of M4 and M3. The black arrows indicate full closure of the fenestration. Note in chain B of repeats #2 and #4 there is no stepwise transition or closure of the fenestration due to presence of a lipid within the fenestration (lipid clog). (**B**) A similar sequence of structural transitions occurs when the POPC bilayer is stretched at a lower pressure of -20 bar. (**C**) A thinner DLPC bilayer fails to drive these structural transitions suggesting that hydrophobic mismatch alone is not sufficient.

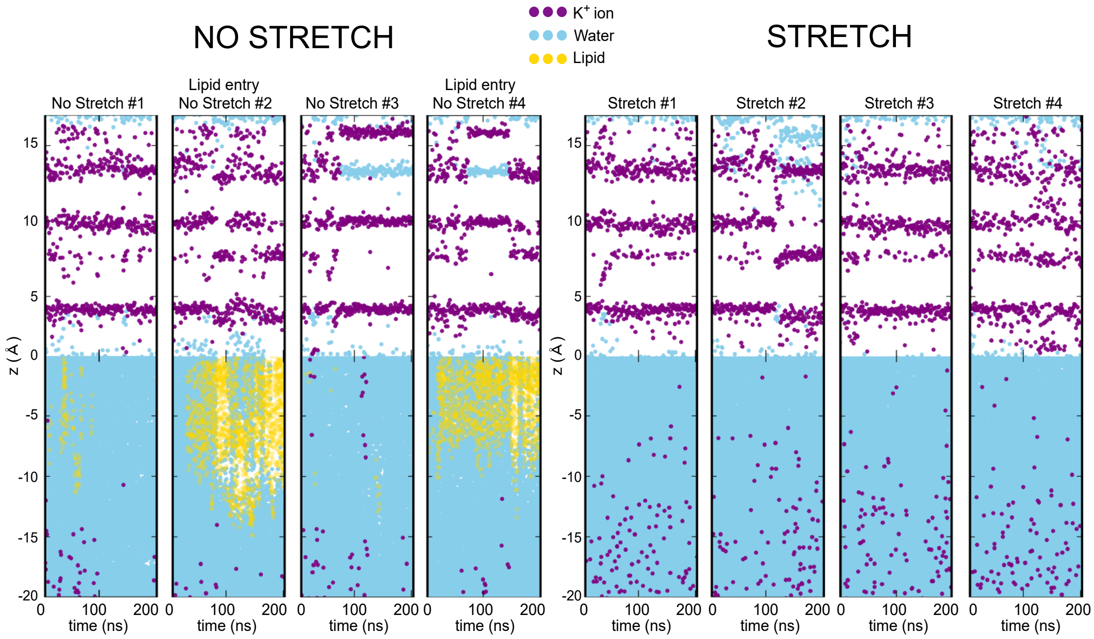

Figure S5. Related to Figure 6.

Effects of membrane stretch on the selectivity filter and hydration of the inner pore cavity. Left: Occupancy along the axis of the pore and inner cavity for ions (purple), water (cyan) and lipid tails (yellow) shown against time of the four independent 200 ns simulations performed in the absence of membrane stretch. Transient dewetting occurs and correlates with the presence of lipid tails (yellow dots) penetrating the cavity, but only in two of the four simulations. Right: As in left panel, but in the presence of membrane stretch (-50 bar). Note the cavity remains fully hydrated in all four simulations (see Figure 6).
