# Supplementary material for: Bilayer-Mediated Structural Transitions Control Mechanosensitivity of the TREK-2 K2P Channel
Source: Structure. 2017 May 2;25(5):708–718.e2. doi: 10.1016/j.str.2017.03.006 (PMC5415359; doi:10.1016/j.str.2017.03.006)
Supplement: Document S2. Article plus Supplemental Information [file mmc2.pdf]

Structure

Article

Bilayer-Mediated Structural Transitions Control Mechanosensitivity of the TREK-2 K2P Channel

Graphical Abstract

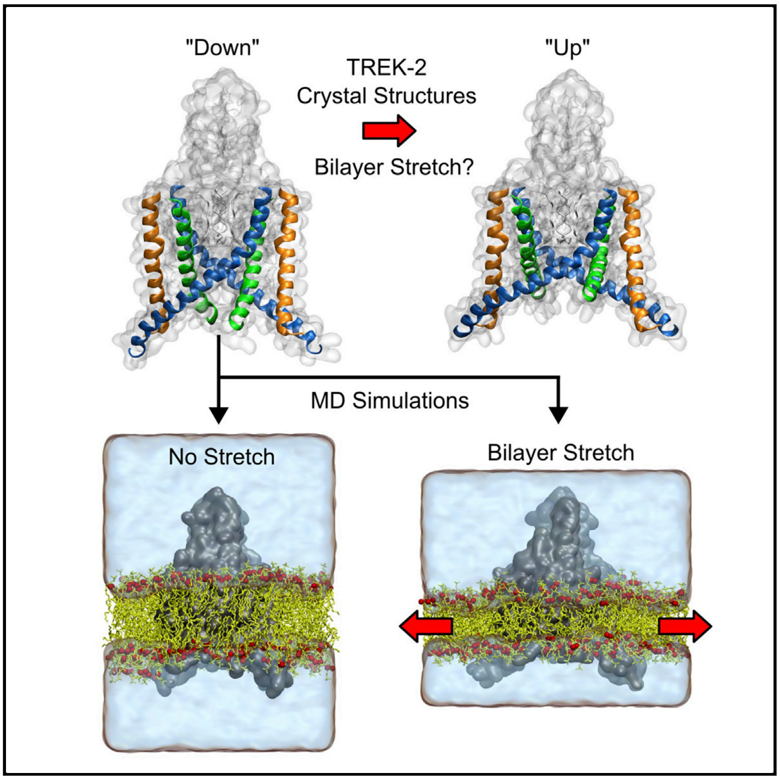

Highlights

- Mechanogating of TREK-2 involves movement from the down to up conformation
- Simulations sample a wide range of mechanosensitive K2P channel structures
- Changes in the pressure profile and state-dependent lipid interactions play a key role
- Lipid block of the inner pore does not mediate stretch activation

Authors

Prafulla Aryal, Viwan Jarerattanachat, Michael V. Clausen, ..., Thomas Baukrowitz, Mark S.P. Sansom, Stephen J. Tucker

Correspondence

mark.sansom@bioch.ox.ac.uk (M.S.P.S.), stephen.tucker@physics.ox.ac.uk (S.J.T.)

In Brief

Aryal et al. use MD simulations to understand stretch activation of the mechanosensitive K2P channel, TREK-2. They demonstrate how a change in forces within the bilayer drives movement between different conformations, and investigate the role that lipids play in this process.

Aryal et al., 2017, Structure *25*, 708–718
May 2, 2017 © 2017 The Authors. Published by Elsevier Ltd.
http://dx.doi.org/10.1016/j.str.2017.03.006

CellPress

Structure
Article

Bilayer-Mediated Structural Transitions Control Mechanosensitivity of the TREK-2 K2P Channel

Prafulla Aryal,[1,2,3,6] Viwan Jarerattanachat,[1,2] Michael V. Clausen,[1] Marcus Schewe,[4] Conor McClenaghan,[1,3] Liam Argent,[2,3] Linus J. Conrad,[1,3] Yin Y. Dong,[5] Ashley C.W. Pike,[5] Elisabeth P. Carpenter,[3,5] Thomas Baukrowitz,[4] Mark S.P. Sansom,[2,3,*] and Stephen J. Tucker[1,3,7,*]
[1]Clarendon Laboratory, Department of Physics, University of Oxford, Oxford OX1 3PU, UK
[2]Department of Biochemistry, University of Oxford, Oxford OX1 3QU, UK
[3]OXION Initiative in Ion Channels and Disease, University of Oxford, Oxford OX1 3PT, UK
[4]Department of Physiology, University of Kiel, 24118 Kiel, Germany
[5]Structural Genomics Consortium, University of Oxford, Oxford OX3 7DQ, UK
[6]Present address: Department of Physiology and Biophysics, University of Colorado Anschutz Medical Center, Denver, CO 80045, USA
[7]Lead Contact
*Correspondence: mark.sansom@bioch.ox.ac.uk (M.S.P.S.), stephen.tucker@physics.ox.ac.uk (S.J.T.)
http://dx.doi.org/10.1016/j.str.2017.03.006

SUMMARY

The mechanosensitive two-pore domain (K2P) K^+ channels (TREK-1, TREK-2, and TRAAK) are important for mechanical and thermal nociception. However, the mechanisms underlying their gating by membrane stretch remain controversial. Here we use molecular dynamics simulations to examine their behavior in a lipid bilayer. We show that TREK-2 moves from the "down" to "up" conformation in direct response to membrane stretch, and examine the role of the transmembrane pressure profile in this process. Furthermore, we show how state-dependent interactions with lipids affect the movement of TREK-2, and how stretch influences both the inner pore and selectivity filter. Finally, we present functional studies that demonstrate why direct pore block by lipid tails does not represent the principal mechanism of mechanogating. Overall, this study provides a dynamic structural insight into K2P channel mechanosensitivity and illustrates how the structure of a eukaryotic mechanosensitive ion channel responds to changes in forces within the bilayer.

INTRODUCTION

Mechanosensitive ion channels rapidly translate mechanical forces within the cell membrane into changes in electrical activity. They therefore underlie a wide range of important physiological processes such as touch, hearing, balance, and the sensation of pain, as well as our ability to regulate blood pressure and other fundamental processes such as osmotic homeostasis (Ranade et al., 2015; Sukharev and Sachs, 2012). However, there are numerous different types of ion channels embedded within the cell membrane, and it is unclear why some channels are particularly sensitive to mechanical stimuli while others are not (Anishkin et al., 2014; Teng et al., 2015). Much of our current understanding of mechanosensitive channels comes from a combination of structural, biophysical, functional, and computational studies of the prokaryotic channels, MscS and MscL (Booth and Blount, 2012). These channels directly sense forces within the lipid bilayer and are gated open by changing their shape and expanding in response to a change in these lateral forces (i.e., bilayer tension). This mechanism of mechanosensitivity is often referred to as the "force from lipid" paradigm (Martinac et al., 1990; Teng et al., 2015).

TREK-2 is a eukaryotic mechanosensitive ion channel that belongs to the two-pore domain (K2P) family of K^+-selective channels (Enyedi and Czirjak, 2010; Renigunta et al., 2015; Sepulveda et al., 2015). However, TREK-2 along with the related TREK-1 and TRAAK channels, are the only members of this family which exhibit mechanosensitivity (Brohawn et al., 2014b). They are widely expressed in both the central and peripheral nervous system and, in addition to their mechanosensitivity, are regulated by a wide range of other biological, chemical, and physical stimuli including pH, temperature, lipids, and anesthetics. Such "polymodal" regulation allows these channels to integrate many different signaling pathways to control membrane excitability including several nociceptive pathways (Mathie and Veale, 2015; Pereira et al., 2014). Crystal structures have now been determined for all three of these mechanosensitive K2P channels as well as for the non-mechanosensitive channel, TWIK-1 (Brohawn et al., 2012; Dong et al., 2015; Miller and Long, 2012). Thus, in addition to their potential as targets for therapeutic drug design, these channels also represent an excellent model system to explore the structural basis of mechanosensitivity.

Early functional studies suggested that K2P channels lack a classical bundle-crossing gate and gate primarily within the selectivity filter (Bagriantsev et al., 2011; Piechotta et al., 2011; Rapedius et al., 2012; Zilberberg et al., 2001). Subsequent crystal structures of TRAAK with the transmembrane (TM) helices in different orientations provided the first structural insights into their gating mechanism by revealing two main conformational states, both of which lacked a bundle-crossing gate (Brohawn et al., 2013, 2014a; Lolicato et al., 2014). These structures

 © 2017 The Authors. Published by Elsevier Ltd.
This is an open access article under the CC BY license (http://creativecommons.org/licenses/by/4.0/).

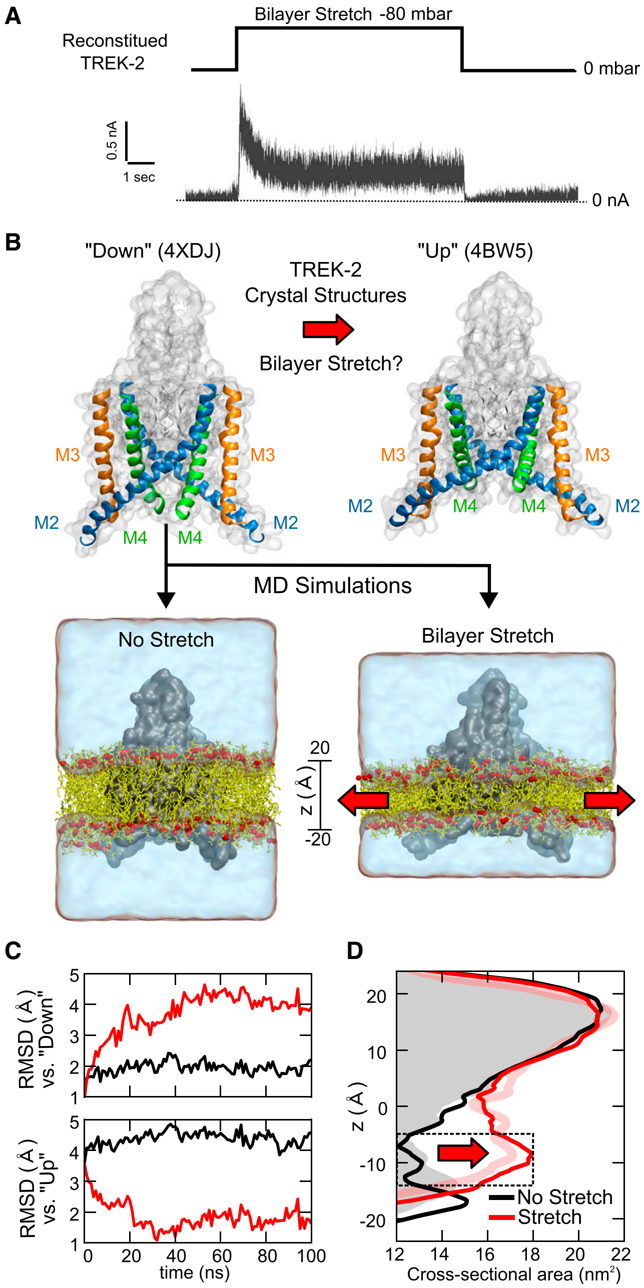

Figure 1. Bilayer Stretch Induces Movement of TREK-2 from the Down State toward the Up State to Produce a Change in Cross-Sectional Area

(A) Intrinsic mechanosensitivity of the purified core TM domains of TREK-2 reconstituted into a planar lipid bilayer. Example of currents recorded at +80 mV in response to a pressure jump of −80 mbar.

(B) Top: a model for K2P channel mechanosensitivity based on comparison of the down and up state crystal structures of TREK-2. Bottom: to test this, we used MD simulations to examine whether membrane stretch can induce a down to up conformational transition. Simulation of membrane stretch (i.e., increased tension) involves an increase in the xy plane of the bilayer (red arrows) to increase the area/lipid (see also Figure S1A).

(C) Top: comparison of Cα RMSD for the TM helices (M1-M4) of TREK-2 against the down state crystal structure during stretch and unstretched simulations. The structure moves away from the down state when stretched (red), but remains close to the down state when unstretched (black). Bottom: similar RMSD comparison against the up state showing rapid stretch-induced movement toward an up-state-like conformation.

(D) Increase in cross-sectional area upon membrane stretch. The dotted box indicates the region of greatest structural change. The cross-sectional area of stretched structure is very similar to the up state crystal structure (PDB: 4BW5; pink line). Unstretched simulations remain close to the down state structure (PDB: 4XDJ; shaded gray area). The z axis is centered on the middle of the bilayer.

suggested that movement between these "up" and "down" states might gate the channel in response to membrane stretch. One of these studies proposed that stretch activation involved movement from the down to the up state (Brohawn et al., 2014a). This was also supported by a parallel study where we solved the structure of TREK-2 in similar up and down conformations (Dong et al., 2015). Moreover, we also identified a binding site for the state-dependent inhibitor norfluoxetine (NFx) located within the side-portals or "fenestrations" which only exist in the down state of the channel. Thus by examining the state dependence of NFx inhibition we were also able to propose a model for stretch activation of TREK-2, which involves movement from the down to the up state (Dong et al., 2015; McClenaghan et al., 2016). However, another study of mutant TRAAK channel structures concluded that the down state represents the stretch-activated state (Lolicato et al., 2014), thus highlighting the inherent difficulties involved in functional annotation of ion channel crystal structures determined in the absence of their native bilayer environment.

In this study, we have used molecular dynamics (MD) simulations to examine the role the lipid bilayer plays in stretch activation of TREK-2. We demonstrate that stretching the bilayer leads to a structural switch from the down to the up conformation, and we have identified the global and local rearrangements within the protein as well as interactions with the bilayer that enable this rapid transition. We also show that lipids and other small molecules can directly influence these movements, and have obtained both structural and functional evidence about the role of the selectivity filter and inner pore in this process. Overall, these results now present a dynamic model of how K2P channels are regulated by changes in membrane tension.

RESULTS AND DISCUSSION

TREK-2 Is Intrinsically Mechanosensitive

We have previously shown that the truncated "TM/Pore domain only" construct used to obtain crystal structures TREK-2 (residues 67–340) retains its mechanosensitivity when expressed in *Xenopus* oocytes (Dong et al., 2015). Comparable results have also been shown for analogous truncated TREK-1 and TRAAK channel proteins when reconstituted into lipid bilayers (Brohawn et al., 2014b). We therefore sought to confirm that this mechanosensitive phenotype is also intrinsic to the TREK-2 construct we simulate in this study. The "crystal construct" TREK-2 protein was therefore expressed and purified as described previously (Dong et al., 2015) and currents recorded after reconstitution into a lipid bilayer. Negative pressure (−80 mbar) was used to increase tension within the membrane. This resulted in a fully reversible 16.3 ± 3.2 fold-increase in channel currents recorded at +80 mV (n = 18) (Figure 1A) and confirms that, like TREK-1 and

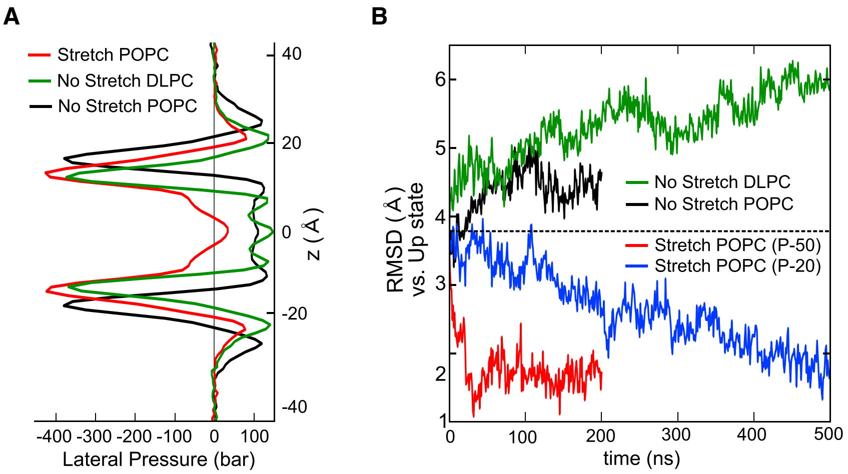

Figure 2. A Role for Changes in the Transmembrane Pressure Profile

(A) Lateral pressure profiles calculated for the bilayers used in this study. Black: unstretched POPC bilayer. Red: POPC bilayer stretched at −50 bar. Note the reduction in positive pressures within the core of the bilayer. The stretched bilayer has the same thickness as an unstretched DLPC bilayer (~31 Å), but the DLPC profile (green) retains positive pressures within the core.

(B) RMSD comparison with the up state crystal structure. Hydrophobic mismatch in the thinner DLPC bilayer (green) is unable to drive movement toward the up state even after 500 ns, instead the structure begins to diverge from the up state (see also Figure S2B). By contrast, low levels of stretch at −20 bar for a similar period produce movement toward the upstate (blue). The changes produced by stretch at −50 bar and no stretch over 200 ns are shown for comparison.

TRAAK, the TM/Pore domains of TREK-2 retain an intrinsic functional response to membrane tension.

Simulation of Bilayer Stretch

Previous MD studies of prokaryotic mechanosensitive channel crystal structures have shown how simulating changes in lateral tension within the bilayer can provide important insights into their mechanogating (Gullingsrud and Schulten, 2004; Kocer, 2015). We therefore examined whether similar computational approaches could be applied to study the behavior of TREK-2 within the membrane (Figure 1B). These MD protocols simulated an increase in membrane tension by increasing the xy plane of the bilayer. We therefore adapted these methods to perform a series of 100 ns all-atom MD simulations at various lateral pressures down to −50 bar on the TREK-2 down state crystal structure (4XDJ) embedded in a 1-palmitoyl-2-oleoyl-*sn*-glycero-3-phosphocholine (POPC) bilayer (see Figure 1B and STAR Methods for details). Consistent with these previous studies, we observed that decreasing lateral pressures (i.e., increasing tensions) led to a stable increase in the area per lipid within the first 10–20 ns when compared with a control no-stretch simulation (Figure S1A).

To determine whether these changes in the bilayer induced a movement from the down to the up state we compared the root-mean-square deviation (RMSD) of the Cα atoms for the TM helices (M1-M4) of the channel against both the down and up state crystal structures of TREK-2. This revealed that TREK-2 remained very close to the down state conformation during the unstretched simulation, but that increasing tension induced a conformational change toward the up state (Figure S1A). This effect was most pronounced at P-50 (i.e., −50 bar), where the RMSD comparison (Figure 1C) revealed a rapid stepwise movement away from the down state toward a conformation very similar to the up state. Importantly, these structural changes were observed at much lower simulated pressures (P-50) than the near membrane-lytic tensions required to produce structural changes in simulations of MscL. Furthermore, even lower simulated pressures could also produce this effect, albeit over a longer timescale (see below, e.g., Figure 2B). This is therefore consistent with the lower pressures required for functional activation of TREK-2. Overall this demonstrates that increasing lateral tension quickly expands the bilayer, and that these changes produce a down to up conformational change consistent with our proposed model for mechanogating of K2P channels (Figure 1B) (Dong et al., 2015; McClenaghan et al., 2016). In further agreement with this model, the up state structure (4BW5) exhibits a highly stable conformation when subjected to the P-50 stretch protocol (Figure S1B).

Bilayer Stretch Induces a Change in Cross-Sectional Area

This change in conformation of the down state toward the up state during our simulations is due to conformational changes located in the lower (i.e., cytoplasmic) half of the protein. This is a likely consequence of the fact that mechanosensitive K2P channels must maintain the structural integrity of their K^+-selective filter in the upper half of the protein during mechanogating. Nevertheless, this expansion in the lower half produces an increase in cross-sectional area of ~4–5 nm^2, very similar to that predicted by comparison of the crystal structures (Figure 1D), and by functional studies (Maksaev et al., 2011). These changes occurred within ~50 ns using the highest (but non-lytic) P-50 stretch protocol.

Changes in the Lateral Pressure Profile

The complex nature of the forces which underlie self-assembly of a lipid bilayer produce an inhomogeneous profile of forces that change with depth across the membrane. In particular, an area of high tension is found just below the polar headgroups that is counterbalanced by areas of positive pressure between the head groups and within the core of the bilayer (Cantor, 1997, 1999). These forces can be calculated and examined by MD simulation, and the resulting lateral "pressure profile" shown to change upon membrane stretch. Importantly, changes in this profile can have a fundamental effect on the properties of proteins embedded within the bilayer, in particular, mechanosensitive ion channels (Gullingsrud and Schulten, 2004; Ollila et al., 2009; Yoo and Cui, 2009; Zhang et al., 2016).

To investigate the effect of changes in the pressure profile on the down to up structural transition, we first ran ms duration

coarse-grained MD simulations for both a stretched and unstretched POPC bilayer and calculated the pressure profile in the absence of embedded protein (see STAR Methods for details). Consistent with previous reports, these profiles demonstrate the non-uniform profile of these forces as a function of depth (Figure 2A), and also reveal how membrane stretch alters the bilayer properties. In particular, stretch results in a decrease in the positive pressures within the core of the bilayer that is offset by an increase in negative pressures at the interfacial region, along with a decrease in bilayer thickness from ~36 to ~31 Å (Figure 2A). Changes in the pressure profile may therefore influence the ability TREK-2 to move between the up and down conformations, and some of the largest changes in the profile (Figure 2A) appear to correlate with the sections of the channel which expand most upon membrane stretch (Figure 1D). However, the membrane also becomes thinner and this will produce hydrophobic mismatch with the TM helices. It is therefore possible that this mismatch may also contribute to the conformational changes we observe (Bavi et al., 2016b; Elmore and Dougherty, 2003; Perozo et al., 2002).

To examine whether a change in bilayer thickness alone is sufficient to drive this structural transition, we performed atomistic simulations of the TREK-2 down state in an unstretched 1,2-dilauroyl-*sn*-glycero-3-phosphocholine (DLPC) bilayer. The shorter acyl tails of the DLPC lipids result in a similar thickness to a POPC bilayer (stretched at −50 bar), but the overall pressure profile of this bilayer is different because it retains the regions of positive pressure typically found within the core of an unstretched bilayer (Figure 2A). However, even after 500 ns of simulation in the thinner DLPC bilayer we observed no movement toward the up state (Figure 2B), nor any increase in cross-sectional area (Figure S2). This suggests that a change in bilayer thickness and hydrophobic mismatch alone is not sufficient to drive this structural transition. By contrast, a similar-length (500 ns) simulation of membrane stretch at a low lateral pressure (−20 bar) in a POPC bilayer was able to induce movement toward the up state (Figure 2B), suggesting that changes in the pressure profile at the lipid-protein interface are more important. To further examine this idea we also calculated profiles for both stretched and unstretched POPC bilayers in the presence of embedded TREK-2 protein. These pressure profiles are more complex (Figure S2C), but overall they reveal similar changes upon membrane stretch, especially the reduction in positive pressures within the core of the bilayer. Overall, these results suggest that movement from the down to the up state is due to changes in the pressure profile around the protein.

Sequential Movements Underlie the Expansion of TREK-2

By examining multiple simulations, we observed that the expansion of TREK-2 is produced by a combination of global and local changes involving sequential movements in the lower sections of the M2, M3, and M4 helices which bend and reorganize themselves in response to membrane stretch (Figure S3). To understand these movements more accurately, we measured three distances which define interactions between these three helices: (1) the "Fenestration" distance between Gly^{324} on M4 and Pro^{198} on M2 of the adjacent chain, (2) the "Zipper" between Trp^{326} on M4 and Arg^{237} on M3 of the same chain, and (3) the "Expansion" between Met^{322} on M4 and Gly^{212} on M2 of the same chain (Figure 3A). Representative plots of these distances during a stretch simulation are shown in Figure 3B. Initially, M2 tilts and slightly narrows the fenestration, but before any further closure can occur, the lower sections of M4 and M3 have to "unzip" which involves a major reorientation of Trp^{326} on M4. This unzipping then permits an expansion between M4 and M2, and reorientation of Phe^{244} to fill the space created during the expansion. Finally, this allows the lower part of M4 to move up and seal the fenestration. Repeated simulations reveal that these movements occur independently and stochastically for the two individual chains of TREK-2 (Figure 3B), although unzipping always precedes expansion and closure of the fenestration (Figure S4). The relevance of these movements is supported by our previous functional studies which demonstrate that mutation of the key residues we have identified (Trp^{326}, Arg^{237}, Met^{322}, and Phe^{244}) markedly reduces TREK-2 mechanosensitivity (Dong et al., 2015).

Correlation plots of these three distances compared with equivalent distances in the TREK-2 down and up crystal structures are shown in Figure 3C. These plots reveal that during the course of an unstretched simulation the fenestration remains open, with M4 interacting closely with the adjacent M2 and M3 of the same chain, thereby sampling conformations very similar to the down state crystal structure. However, when stretched, the fenestration closes and the channel then samples conformations very similar to the TREK-2 up state crystal structure. This down to up transition occurred in all of the four separate simulations except where the presence of a lipid tail trapped between the M2 and M4 helices resulted in incomplete closure of a fenestration (see below for discussion and also Figure S4).

Lack of Stretch-Induced Changes in the Non-mechanosensitive TWIK-1 Channel

It is possible that any channel structure embedded within a bilayer might change in response to membrane stretch. Therefore, to assess the specificity of our results, we repeated our stretch protocol using the crystal structure of the homologous, but non-mechanosensitive K2P channel, TWIK-1 (Miller and Long, 2012). We found that even the P-50 stretch protocol for 200 ns produced no change in the cross-sectional area of TWIK-1 and little structural divergence from the initial conformation (Figures 4A and 4B). This suggests that these stretch-induced conformational changes are specific to mechanosensitive K2P channels.

TWIK-1 differs from the mechanosensitive K2Ps in its shape, structure, and flexibility. For example, the cross-sectional area of the TREK-2 down state is more asymmetric than TWIK-1 (cf. Figures 1D and 4B), and TREK-2 has longer amphipathic tips connecting M2 and M3. TWIK-1 also has a highly stable amphipathic C helix at the end of M4 (Aryal et al., 2014, 2015a; Miller and Long, 2012). Furthermore, whereas TREK-2 possesses >20 glycine residues within the core M2-M4 region, TWIK-1 has only 8. Therefore, it is possible that a combination of these structural differences allows TREK-2 to exhibit a greater flexibility within the membrane.

MD Simulations and Crystal Structures Sample the Same Conformational Landscape

Quite remarkably for a class of human ion channels, over 12 different crystal structures now exist for the three known

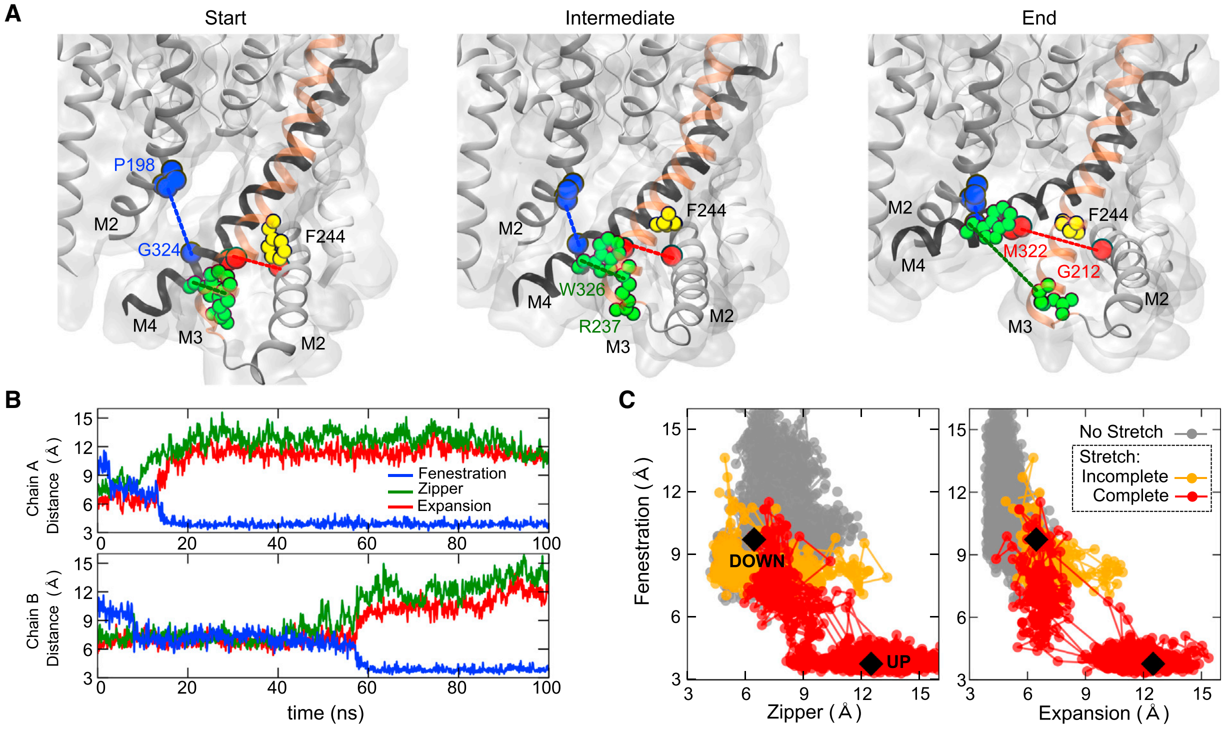

Figure 3. Sequential Rearrangement of the TM Helices during Membrane Stretch

(A) Movement of TMs measured as a change in three distances: (1) "Fenestration" between G324 and P198 of the adjacent subunit (blue), (2) "Zipper" between W326 and R237 (green), and (3) "Expansion" between M322 and G212 (red). These changes are shown at the start, during, and end of 100 ns stretch simulation. Note unzipping of interactions between M4 and M3 requires reorientation of W326 (green) on M4; also F244 (yellow) then rotates to fill the space created by the M2-M4 expansion.

(B) Change in these distances in separate chains of TREK-2 during a stretch simulation. Note the sequence: an initial contraction of the fenestration followed by unzipping, expansion, and then full closure of the fenestration.

(C) Correlation plot comparing the change in fenestration and zipper distances during an unstretched (gray) and stretched (red) simulation sampled every 1 ns. Distances in the TREK-2 crystal structures shown as black diamonds. Within the unstretched bilayer TREK-2 samples conformations close to the down state crystal structure (PDB: 4XDJ) and does not approach the up state conformation (PDB: 4BW5). When stretched (red), the structure samples conformations similar to the up state. In some cases incomplete closure of the fenestration occurs (orange) due to obstruction by lipid tails (see also Figures 6 and S5). The right-hand panel shows a similar comparison of fenestration and expansion distances for both stretch and unstretched simulations.

mechanosensitive K2P channels and represent a variety of up- and down-like conformations (Figure 4C). Our results demonstrate that bilayer stretch favors a switch from the down to the up state. But such movement also requires the channel to transition through a variety of intermediate conformations. We therefore compared the fenestration and expansion distances in these different crystal structures to those which occur in TREK-2 during our simulations.

These plots (Figure 4D) show that, in the unstretched bilayer, TREK-2 samples a variety of conformational states similar to all of the known "down-like" crystal structures, but does not approach any conformations similar to the up state structures. By marked contrast, the channel moves away from the down state when stretched, and samples conformations representative of the various up state crystal structures (Figure 4D). This observation not only helps validate our MD simulations and stretch protocols, but also highlights the relevance of the many different crystal structures that have been identified for these mechanosensitive channels.

Norfluoxetine Prevents Closure of the Fenestration

As a further control, we repeated the stretch simulations with NFx bound within the fenestrations. The NFx-bound crystal structure of TREK-2 (4XDL) was used as a guide to place the drug in its binding site and the structure then simulated with and without membrane stretch. As predicted from our previous functional studies (Dong et al., 2015; McClenaghan et al., 2016), the presence of NFx in its binding site in the upper part of the fenestration (Figure 4E) interfered with closure of the interface between M4 and adjacent M2, thus slowing movement toward the up state (Figure 4F). This is also consistent with our previous functional studies which show that NFx markedly slows the kinetics of stretch activation (Dong et al., 2015).

Influence of Bound Lipids

Similar to the ability of NFx to prevent full closure of the fenestration, our simulations also reveal that state-dependent binding of surrounding lipids can affect these structural transitions. In particular, the occupancies of two different binding sites appear

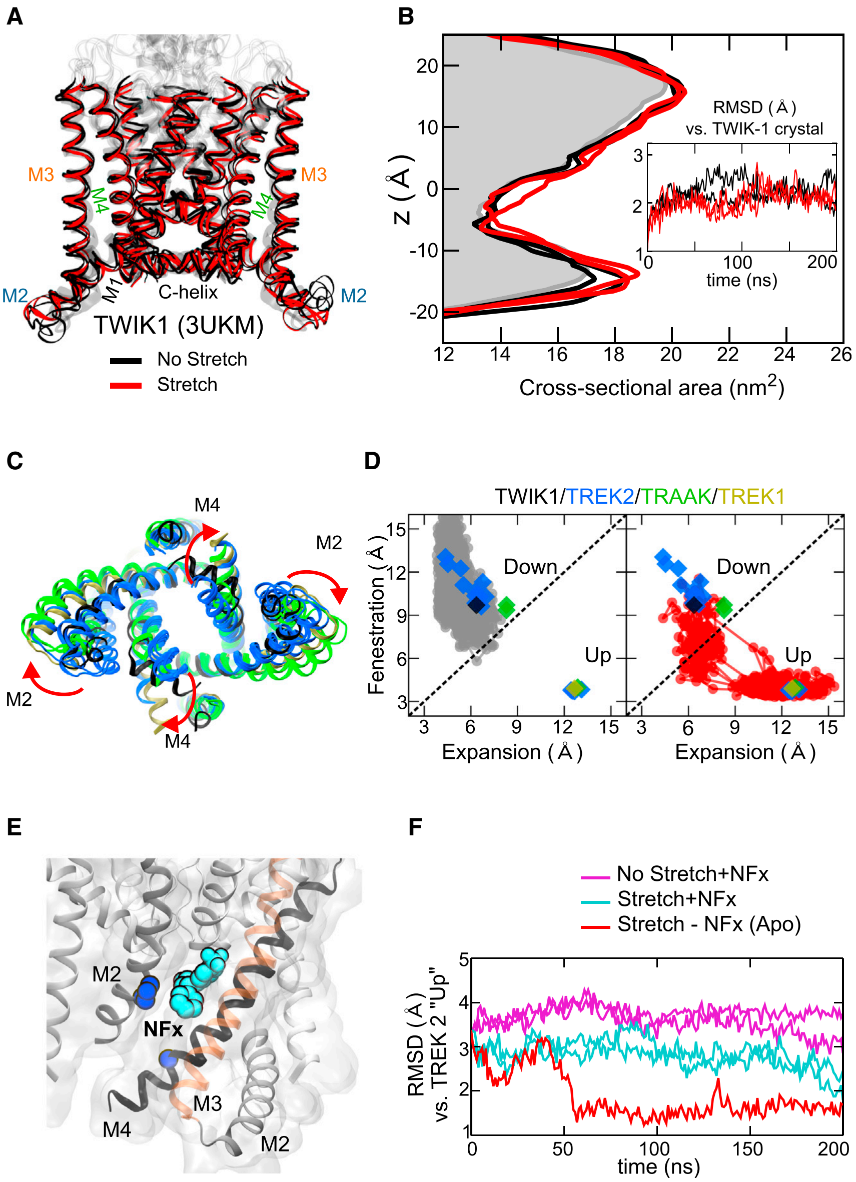

Figure 4. Specificity of the Stretch Simulations

(A) Overlaying TWIK-1 structures simulated for 200 ns in the presence or absence of membrane stretch (−50 bar) reveals no change in structure. The starting structure is shown in gray.

(B) No change in cross-sectional area in response to membrane stretch. The shaded gray area represents starting structure. Inset: comparison of Cα RMSD for M1-M4 helices against the TWIK-1 crystal structure (3UKM) with or without stretch. Overall, there is no change in response to stretch, even at −50 bar.

(C) Overlay of different up and down state crystal structures available for TWIK-1, TREK-1, TREK-2, and TRAAK demonstrating the wide variety of conformational states. Colors are as shown in (D).

(D). Correlation plots of fenestration versus expansion distances for TREK-2 simulations with or without stretch. Equivalent distances in the known crystal structures are plotted as colored diamonds. In the absence of stretch (gray dots) the structure samples conformational states similar to the down state crystal structures of both TREK and TRAAK. But when stretched (red dots), the structure quickly samples conformations similar to the many up state crystal structures. Dots represent sampling every 1 ns during the simulation.

(E) Structure of TREK-2 in the down state showing location of norfluoxetine (NFx; cyan) within its binding site in the upper fenestration. G324 on M4 and P198 on M2 highlighted blue.

(F) RMSD comparison against the up state crystal structure for unstretched (purple) and stretched simulations (cyan), both had NFx located within its binding sites at the start of the simulation. The control stretch simulation is identical but with the NFx deleted (Apo, red). The presence of NFx within the fenestration binding site prevents rapid movement toward the up state conformation upon stretch.

to be highly correlated with these structural transitions. We observed that lipids were closely associated with the external surface of TREK-2 near the fenestrations in all of the unstretched simulations. However, when stretched, this binding site disappears, indicating a state-dependent effect (Figure 5A). Interestingly, we also found that, in the two stretch simulations which did not exhibit a sequential movement of M2/M3/M4 (i.e., the incomplete closure shown in Figures 3C and S4), lipids had moved into the fenestrations. In each case, the lipid head group approached close enough to the lower part of the fenestration that the lipid was able to enter and remain there for the rest of the simulation. To confirm whether this "lipid clog" prevented closure, we returned to one of these particular simulations, paused it, and deleted the obstructing lipid. Upon restarting the simulation, the fenestration closed immediately and the protein moved toward the up state preventing further lipid binding at this site (Figure 5A).

We also observed a second state-dependent site within a groove formed by the amphipathic sections connecting the lower tips of M2 and M3 (Figure 5B). These tips are one of the unique features of the tandem-pore K2P channels and are slightly longer in the mechanosensitive TREK/TRAAK channels. In the absence of stretch, this hydrophobic groove interacted with lipid tails throughout the simulation. However, upon stretch, lipid occupation of this site reduced and the groove closed after rearrangement of the tips within the membrane (Figure 5B).

Overall these results are consistent with the "force from lipid" principle of mechanosensitivity, which predicts that stretch leads to a lower density of lipids around the protein and the consequential changes in the lateral pressure profile (Anishkin et al., 2014; Teng et al., 2015). The calculation of lipid-protein interaction free energies in these two states is tempting, but remains challenging because of the slow convergence of the underlying simulations (Domanski et al., 2016). Nevertheless, our results show that state-dependent lipid-protein interactions can influence the movement of TREK-2 and thus may share some

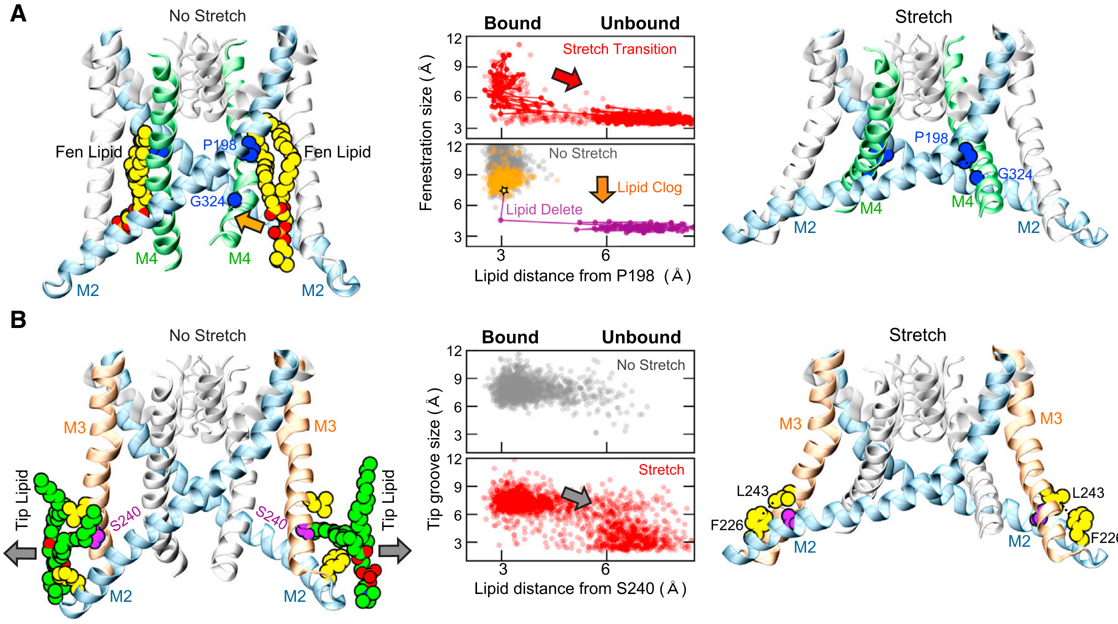

Figure 5. State-Dependent Binding of Lipids Influences Movement of the TM Helices

(A) Left: without stretch, lipids interact with the groove between M4 and M2. Examples of interacting lipids shown as yellow spheres. Right: when stretched, the fenestration closes (P198 and G324 in blue). However, in rare cases the head group can move toward the fenestration (orange arrow) allowing lipid tails to clog the fenestration and prevent closure. Middle: Fenestration distances (P198-G324) during a stretched simulation are plotted against the minimum distance of lipid tails from P198 within the fenestration. During stretch (red), lipids initially occupy this site ("Bound," i.e., come within 4 Å of P198), but upon closure of the fenestration this site is no longer available. Without stretch (gray) the fenestration remains open and lipids are bound within this groove. In some stretch simulations (orange dots) full closure does not occur due to "lipid clog." Deletion of the obstructing lipid (purple) allows rapid closure of the fenestration.

(B) Left: a second stretch-dependent lipid binding site between the tips of M2/M3. Without stretch, a hydrophobic groove is present between F226 on M2 and L243 on M3 (yellow). A snapshot is shown of lipid tails (green) within this groove during a non-stretch simulation. The lipid moves away (gray arrow) upon stretch. Right: after stretch, lipid occupancy decreases and the tips rotate allowing the groove to close. Middle: plot of the tip groove size (i.e., distance between F226 and L243) against the minimum distance of a lipid tail from S240 (purple) within this groove. Without stretch, lipids bind freely with occasional exchange (gray dots), but when stretched lipid tails move away from this site (gray arrow), the tips rearrange and the groove closes.

possible similarities with the so-called "lipid moves first" model of mechanosensitivity (Pliotas et al., 2015). The lipid moves first model is derived from the "force from lipid principle," but proposes that changes in the lipid occupancy of hydrophobic grooves and pockets influences the ability of the channel to transition between different conformations within the membrane. However, it has also been proposed that stored elastic energy within membrane proteins (Malcolm et al., 2015) and lipid-protein interactions with amphipathic helices (Bavi et al., 2016a) influence conformational changes within the bilayer. Therefore, the relevance of these different processes to the energetics of the up-down transition in TREK-2 remains to be determined, but these mechanisms all share a degree of mechanistic similarity and are unlikely to prove mutually exclusive.

Functional Consequences of Membrane Stretch

Although two conflicting structural models for mechanoactivation of K2P channels have been proposed, our simulations now provide strong support for the stretch-mediated down to up model. However, the reasons why this stretch-induced transition produces a more conductive state remain unclear, and several different models have also been suggested. One study has proposed that the down state is permanently non-conductive because lipid tails enter the fenestration to directly occlude the inner pore and K^+ permeation (Brohawn et al., 2014a), whereas several other studies have demonstrated the role of the selectivity filter in mechanogating (Bagriantsev et al., 2011; Piechotta et al., 2011; Schewe et al., 2016). In addition, it has also been proposed that "dewetting" of the inner pore may play a role in K2P channel gating (Aryal et al., 2014, 2015a). We therefore investigated our simulations to explore these different ideas.

First, we examined the effect of stretch on the selectivity filter. Interestingly, we found that in the unstretched simulations K^+ ions and water fluctuated between the S1 and S0 sites (Figures 6A, 6B, and S5). Also of interest, we found that stretch altered these fluctuations to produce a decrease in K^+ occupancy at S0 and an increase at S1 (Figure 6B). This demonstrates that membrane stretch can change ion occupancy within the filter and may therefore be related to the role that ions play in gating the filter of K2P channels (Schewe et al., 2016).

We next examined the status of the inner pore in the stretched and unstretched simulations. Without stretch, TREK-2 remained in a down-like conformation with the side fenestrations open, and in two of the four simulations we found that lipid tails penetrated these gaps (Figure 6A) to induce transient dewetting of the inner cavity similar that observed in TWIK-1 (Aryal et al., 2014, 2015a). By contrast, no dewetting was observed in any of the four stretch simulations where the inner pore exhibits a higher degree of water occupancy (Figures 6C and S5).

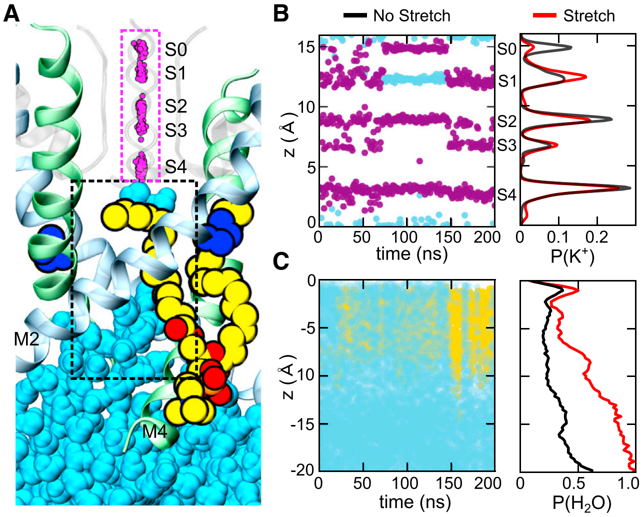

Figure 6. Effects of Membrane Stretch on the Selectivity Filter and the Inner Pore Cavity

(A) Snapshot of the filter and inner cavity during an unstretched simulation. Locations visited by K^+ ions within the S0-S4 binding sites are shown as small purple spheres. Also shown are water molecules (cyan) and an example of a POPC lipid tail penetrating the lower part of the fenestration. Note this type of lipid penetration does not occur in all unstretched simulations (see also Figure S5). P198 on M2 is shown in blue.

(B) Effect of membrane stretch on ion occupancy within the filter. Left: occupancy along the pore axis of ions (purple) and water in the filter for an unstretched simulation. Transitions occur between the S0 and S1 sites in the unstretched simulations (see Figure S5). Right: overall probability of ions in the filter ± stretch. The non-stretched simulations (black line) show increased K^+ occupancy at S0 and reduced occupancy at S1 suggesting how stretch may directly influence the filter gate.

(C) Left: rare example where lipid penetration, via route shown in (A), produces transient dewetting. Presence of water (cyan) and lipid tails (yellow) along the axis of the pore is shown against time. Dewetting correlates with the entry of lipid tails, but does not occur in all unstretched simulations. Right: stretch results in an increase in water density deep within the cavity (red line), but overall the inner pore remains hydrated during the unstretched simulations (see also Figure S5).

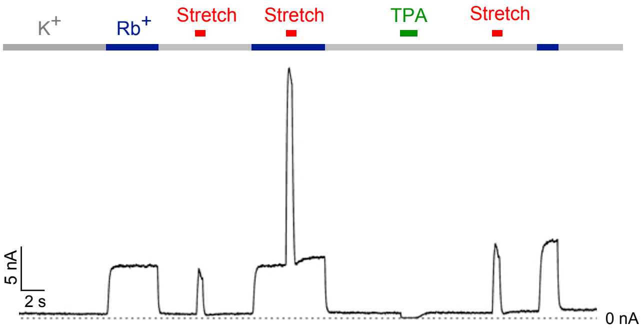

Figure 7. Direct Access of Permeant Ions to the Selectivity Filter in the Absence of Membrane Stretch

Example of a giant excised patch recording for mechanosensitive TREK channels expressed in *Xenopus* oocytes. Currents show alternating activation by membrane stretch (−15 mmHg applied to the patch pipette; red bar) or replacement of intracellular K^+ (gray bar) with Rb^+ (blue bar). Rb^+ as the permeant ion produces direct activation of a voltage-dependent gate within the filter, indicating free access from the cytoplasmic side. Membrane stretch (red bar) also activates channels to a similar extent. When applied together with Rb^+ the effects are synergistic. If lipids directly prevented K^+ permeation (Brohawn et al., 2014a), then Rb^+ would not be able to access the filter in the absence of membrane stretch. Instead, Rb^+ activates the filter gate both before and after membrane stretch, indicating free access to the filter. Inhibition of channel activity by 100 μM tetrapentylammonium (TPA) is shown in green as a control. This result clearly demonstrates that direct lipid occlusion of the inner pore does not represent the primary mechanism of mechanogating.

Membrane Stretch Directly Activates the Filter Gate

Although lipid penetration of the fenestrations was observed (e.g., Figure 6A), it did not occur in all of the unstretched simulations and differs from the proposed horizontal entry of lipid tails into the fenestrations of TRAAK (Brohawn et al., 2014a). It is also important to note that the intracellular C-terminal domain attached to the end of M4, but missing in the crystal structures, may also obstruct the entry of lipids via the lower part of the fenestration. Nevertheless, if lipid entry does occur then it clearly has the potential to directly occlude the pore and possibly "gate" the channel closed. But, if direct lipid block of K^+ permeation represents the primary mechanism of TREK/TRAAK channel mechanogating (Brohawn et al., 2014a) then it does not explain why the down state of the channel can be gated open in the absence of membrane stretch (McClenaghan et al., 2016), why mechanoactivation does not affect access of quaternary ammonium ion blockers to their binding site just below the filter (Piechotta et al., 2011), or why the selectivity filter appears to play such a major role in K2P channel gating (Bagriantsev et al., 2011; Piechotta et al., 2011; Rapedius et al., 2012; Zilberberg et al., 2001).

For example, we have recently shown that permeant ions such as Rb^+ activate nearly all K2P channels (including TREK-1, TREK-2, and TRAAK) by directly influencing a voltage-dependent gate within the selectivity filter (Schewe et al., 2016). Therefore we tested the "lipid occlusion" model of mechanogating (Brohawn et al., 2014a) by comparing stretch and Rb^+ activation of TREK-1 channels expressed in *Xenopus* oocytes (Figure 7). These recordings show that channels within the same giant excised patch can be alternately and reversibly activated to the same extent by either intracellular application of Rb^+ and/or membrane stretch (fold-increase by membrane stretch = 10.5 ± 2.5, n = 6; for Rb^+ activation = 9.2 ± 1.3, n = 6). Also, the pressure activation appears markedly enhanced in channels activated by Rb^+.

These effects are not consistent with a lipid occlusion model because intracellular Rb^+ would not be able to access the filter if lipids directly blocked the permeation pathway in the down state. Also, the synergistic effects of Rb^+ and stretch are similar to the functional effects of combining stretch and intracellular pH activation (Kim et al., 2001), thus emphasizing the complex role the filter plays in channel gating. These results do not exclude a potential role for lipids in directly influencing the properties of the pore, but they demonstrate that direct block of the conduction pathway by lipid tails does not represent the principal mechanism of mechanogating. Instead, more complex mechanisms including not only filter gating, but also hydration and electrowetting of the inner cavity may be involved (Aryal et al., 2015b).

Conclusions

These studies demonstrate that the core TM domains of TREK-2 are intrinsically mechanosensitive. From a total of over 6 μs of all-atom MD simulations of K2P channels in lipid bilayers, we show that the structure of the mechanosensitive TREK-2 channel can rapidly expand to switch from the down to the up state upon bilayer stretch. Examining the sequence of events which produce this transition also demonstrates that increased tension quickly decreases the density of lipids surrounding the protein, and that mechanosensitive K2P channels rapidly respond by adopting an expanded conformation within the bilayer. The driving force for this transition also correlates with changes in the TM pressure profile and involves state-dependent changes in the interaction of lipids with the protein. In contrast to the non-mechanosensitive TWIK-1, the mechanosensitive K2P channel structures also exhibit a stretch-dependent flexibility which explains the diversity of crystallographic conformations now solved for these channels. Together, these results are consistent with the force from lipid principle, and also the idea that stretch-dependent changes in lipid-protein interactions affect transitions between different structural states (Pliotas et al., 2015).

We have also examined the potential role of lipids in direct gating of the pore and find that direct lipid occlusion does not represent the principal mechanism of mechanogating. Instead, membrane stretch influences ion occupancy within the filter consistent with its role in mechanogating. However, further studies will be required to understand how the channel can be gated open in the absence of membrane stretch, and how movement between these two states regulates the filter gating mechanism.

Interestingly, recent studies have demonstrated that other structurally distinct mechanosensitive ion channels such as Piezo1 also display an intrinsic mechanosensitivity, thus illustrating their innate ability to act as molecular force transducers (Syeda et al., 2016). It will therefore be interesting to examine whether the mechanisms highlighted in this study can provide further insight into the mechanisms of mechanogating in other classes of eukaryotic ion channels.

STAR★METHODS

Detailed methods are provided in the online version of this paper and include the following:

- KEY RESOURCES TABLE
- CONTACT FOR REAGENT AND RESOURCE SHARING
- EXPERIMENTAL MODEL AND SUBJECT DETAILS
- METHOD DETAILS
  - Computational Methods
  - Electrophysiological Methods
- QUANTIFICATION AND STATISTICAL ANALYSIS
  - Electrophysiology
- DATA AND SOFTWARE AVAILABILITY

SUPPLEMENTAL INFORMATION

Supplemental Information includes five figures and can be found with this article online at http://dx.doi.org/10.1016/j.str.2017.03.006.

AUTHOR CONTRIBUTIONS

All computational and experimental work required for completion of this study was performed by P.A., V.J., M.V.C., M.S., C.McC., L.A., L.J.C., Y.Y.D., and A.C.W.P. Studies were supervised by E.P.C., T.B., M.S.P.S., and S.J.T. The manuscript was written by P.A. and S.J.T. with input from all of the authors.

ACKNOWLEDGMENTS

This work was funded by grants to S.J.T., M.S.P.S., and E.P.C. from the Wellcome Trust and the Biotechnology and Biological Sciences Research Council (BBSRC), and to T.B. from the Deutsche Forschungsgemeinschaft. P.A. holds an OXION Training Fellowship and both L.A. and C.McC. hold OXION studentships funded by the Wellcome Trust. M.V.C. was supported by a Lundbeck Foundation Fellowship and V.J. by a Thai Government Scholarship. Y.Y.D., A.C.W.P., and E.P.C. are also funded by a EU/EFPIA Innovative Medicines Initiative (IMI) Joint Undertaking grant, the Wellcome Trust, and by the Structural Genomics Consortium (a registered charity receiving funds from AbbVie, Bayer Pharma AG, Boehringer Ingelheim, the Canada Foundation for Innovation, Genome Canada, GlaxoSmithKline, Janssen, Lilly Canada, Merck & Co., the Novartis Research Foundation, the Ontario Ministry of Economic Development and Innovation, Pfizer São Paulo Research Foundation-FAPESP, and Takeda.

Received: December 16, 2016
Revised: February 1, 2017
Accepted: March 10, 2017
Published: April 6, 2017

REFERENCES

Anishkin, A., Loukin, S.H., Teng, J., and Kung, C. (2014). Feeling the hidden mechanical forces in lipid bilayer is an original sense. Proc. Natl. Acad. Sci. USA *111*, 7898–7905.

Aryal, P., Abd-Wahab, F., Bucci, G., Sansom, M.S., and Tucker, S.J. (2014). A hydrophobic barrier deep within the inner pore of the TWIK-1 K2P potassium channel. Nat. Commun. *5*, 4377.

Aryal, P., Abd-Wahab, F., Bucci, G., Sansom, M.S., and Tucker, S.J. (2015a). Influence of lipids on the hydrophobic barrier within the pore of the TWIK-1 K2P channel. Channels *9*, 44–49.

Aryal, P., Sansom, M.S., and Tucker, S.J. (2015b). Hydrophobic gating in ion channels. J. Mol. Biol. *427*, 121–130.

Bagriantsev, S.N., Peyronnet, R., Clark, K.A., Honore, E., and Minor, D.L., Jr. (2011). Multiple modalities converge on a common gate to control K2P channel function. EMBO J. *30*, 3594–3606.

Bavi, N., Cortes, D.M., Cox, C.D., Rohde, P.R., Liu, W., Deitmer, J.W., Bavi, O., Strop, P., Hill, A.P., Rees, D., et al. (2016a). The role of MscL amphipathic N terminus indicates a blueprint for bilayer-mediated gating of mechanosensitive channels. Nat. Commun. *7*, 11984.

Bavi, O., Vossoughi, M., Naghdabadi, R., and Jamali, Y. (2016b). The combined effect of hydrophobic mismatch and bilayer local bending on the regulation of mechanosensitive ion channels. PLoS One *11*, e0150578.

Berendsen, H.J.C., Postma, J.P.M., van Gunsteren, W.F., DiNola, A., and Haak, J.R. (1984). Molecular dynamics with coupling to an external bath. J. Chem. Phys. *81*, 3684–3690.

Best, R.B., Zhu, X., Shim, J., Lopes, P.E., Mittal, J., Feig, M., and Mackerell, A.D., Jr. (2012). Optimization of the additive CHARMM all-atom protein force field targeting improved sampling of the backbone phi, psi and side-chain chi(1) and chi(2) dihedral angles. J. Chem. Theor. Comput. *8*, 3257–3273.

Booth, I.R., and Blount, P. (2012). The MscS and MscL families of mechanosensitive channels act as microbial emergency release valves. J. Bacteriol. *194*, 4802–4809.

Brohawn, S.G., del Marmol, J., and MacKinnon, R. (2012). Crystal structure of the human K2P TRAAK, a lipid- and mechano-sensitive K+ ion channel. Science *335*, 436–441.

Brohawn, S.G., Campbell, E.B., and MacKinnon, R. (2013). Domain-swapped chain connectivity and gated membrane access in a Fab-mediated crystal of the human TRAAK K+ channel. Proc. Natl. Acad. Sci. USA *110*, 2129–2134.

Brohawn, S.G., Campbell, E.B., and MacKinnon, R. (2014a). Physical mechanism for gating and mechanosensitivity of the human TRAAK K+ channel. Nature *516*, 126–130.

Brohawn, S.G., Su, Z., and MacKinnon, R. (2014b). Mechanosensitivity is mediated directly by the lipid membrane in TRAAK and TREK1 K+ channels. Proc. Natl. Acad. Sci. USA *111*, 3614–3619.

Brooks, B.R., Brooks, C.L., 3rd, Mackerell, A.D., Jr., Nilsson, L., Petrella, R.J., Roux, B., Won, Y., Archontis, G., Bartels, C., Boresch, S., et al. (2009). CHARMM: the biomolecular simulation program. J. Comput. Chem. *30*, 1545–1614.

Bussi, G., Donadio, D., and Parrinello, M. (2007). Canonical sampling through velocity rescaling. J. Chem. Phys. *126*, 014101.

Cantor, R.S. (1997). Lateral pressures in cell membranes: a mechanism for modulation of protein function. J. Phys. Chem. B *101*, 1723–1725.

Cantor, R.S. (1999). Lipid composition and the lateral pressure profile in bilayers. Biophys. J. *76*, 2625–2639.

de Jong, D.H., Singh, G., Bennett, W.F., Arnarez, C., Wassenaar, T.A., Schafer, L.V., Periole, X., Tieleman, D.P., and Marrink, S.J. (2013). Improved parameters for the martini coarse-grained protein force field. J. Chem. Theor. Comput. *9*, 687–697.

Domanski, J., Hedger, G., Best, R.B., Stansfeld, P.J., and Sansom, M.S. (2016). Convergence and sampling in determining free energy landscapes for membrane protein association. J. Phys. Chem. B. http://dx.doi.org/10.1021/acs.jpcb.6b08445.

Dong, Y.Y., Pike, A.C., Mackenzie, A., McClenaghan, C., Aryal, P., Dong, L., Quigley, A., Grieben, M., Goubin, S., Mukhopadhyay, S., et al. (2015). K2P channel gating mechanisms revealed by structures of TREK-2 and a complex with Prozac. Science *347*, 1256–1259.

Elmore, D.E., and Dougherty, D.A. (2003). Investigating lipid composition effects on the mechanosensitive channel of large conductance (MscL) using molecular dynamics simulations. Biophys. J. *85*, 1512–1524.

Enyedi, P., and Czirjak, G. (2010). Molecular background of leak K+ currents: two-pore domain potassium channels. Physiol. Rev. *90*, 559–605.

Faraldo-Gomez, J.D., Smith, G.R., and Sansom, M.S. (2002). Setting up and optimization of membrane protein simulations. Eur. Biophys. J. *31*, 217–227.

Gullingsrud, J., and Schulten, K. (2004). Lipid bilayer pressure profiles and mechanosensitive channel gating. Biophys. J. *86*, 3496–3509.

Hess, B., Bekker, H., Berendsen, H.J.C., and Fraaije, J.G.E.M. (1997). LINCS: a linear constraint solver for molecular simulations. J. Comput. Chem. *18*, 1463–1472.

Hess, B., Kutzner, C., van der Spoel, D., and Lindahl, E. (2008). GROMACS 4: algorithms for highly efficient, load-balanced, and scalable molecular simulation. J. Chem. Theor. Comput. *4*, 435–447.

Humphrey, W., Dalke, A., and Schulten, K. (1996). VMD: visual molecular dynamics. J. Mol. Graph. *14*, 33–38, 27–38.

Kim, Y., Gnatenco, C., Bang, H., and Kim, D. (2001). Localization of TREK-2 K+ channel domains that regulate channel kinetics and sensitivity to pressure, fatty acids and pHi. Pflugers Arch. *442*, 952–960.

Kocer, A. (2015). Mechanisms of mechanosensing – mechanosensitive channels, function and re-engineering. Curr. Opin. Chem. Biol. *29*, 120–127.

Lolicato, M., Riegelhaupt, P.M., Arrigoni, C., Clark, K.A., and Minor, D.L., Jr. (2014). Transmembrane helix straightening and buckling underlies activation of mechanosensitive and thermosensitive K(2P) channels. Neuron *84*, 1198–1212.

Maksaev, G., Milac, A., Anishkin, A., Guy, H.R., and Sukharev, S. (2011). Analyses of gating thermodynamics and effects of deletions in the mechanosensitive channel TREK-1: comparisons with structural models. Channels *5*, 34–42.

Malcolm, H.R., Blount, P., and Maurer, J.A. (2015). The mechanosensitive channel of small conductance (MscS) functions as a Jack-in-the box. Biochim. Biophys. Acta *1848*, 159–166.

Marrink, S.J., Risselada, H.J., Yefimov, S., Tieleman, D.P., and de Vries, A.H. (2007). The MARTINI force field: coarse grained model for biomolecular simulations. J. Phys. Chem. B *111*, 7812–7824.

Martinac, B., Adler, J., and Kung, C. (1990). Mechanosensitive ion channels of *E. coli* activated by amphipaths. Nature *348*, 261–263.

Mathie, A., and Veale, E.L. (2015). Two-pore domain potassium channels: potential therapeutic targets for the treatment of pain. Pflugers Arch. *467*, 931–943.

McClenaghan, C., Schewe, M., Aryal, P., Carpenter, E.P., Baukrowitz, T., and Tucker, S.J. (2016). Polymodal activation of the TREK-2 K2P channel produces structurally distinct open states. J. Gen. Physiol. *147*, 497–505.

Michaud-Agrawal, N., Denning, E.J., Woolf, T.B., and Beckstein, O. (2011). MDAnalysis: a toolkit for the analysis of molecular dynamics simulations. J. Comput. Chem. *32*, 2319–2327.

Miller, A.N., and Long, S.B. (2012). Crystal structure of the human two-pore domain potassium channel K2P1. Science *335*, 432–436.

Moustakas, D.T., Lang, P.T., Pegg, S., Pettersen, E., Kuntz, I.D., Brooijmans, N., and Rizzo, R.C. (2006). Development and validation of a modular, extensible docking program: DOCK 5. J. Comput. Aided Mol. Des. *20*, 601–619.

Ollila, O.H., Risselada, H.J., Louhivuori, M., Lindahl, E., Vattulainen, I., and Marrink, S.J. (2009). 3D pressure field in lipid membranes and membrane-protein complexes. Phys. Rev. Lett. *102*, 078101.

Pereira, V., Busserolles, J., Christin, M., Devilliers, M., Poupon, L., Legha, W., Alloui, A., Aissouni, Y., Bourinet, E., Lesage, F., et al. (2014). Role of the TREK2 potassium channel in cold and warm thermosensation and in pain perception. Pain *155*, 2534–2544.

Perozo, E., Kloda, A., Cortes, D.M., and Martinac, B. (2002). Physical principles underlying the transduction of bilayer deformation forces during mechanosensitive channel gating. Nat. Struct. Mol. Biol. *9*, 696–703.

Piechotta, P.L., Rapedius, M., Stansfeld, P.J., Bollepalli, M.K., Ehrlich, G., Andres-Enguix, I., Fritzenschaft, H., Decher, N., Sansom, M.S., Tucker, S.J., and Baukrowitz, T. (2011). The pore structure and gating mechanism of K2P channels. EMBO J. *30*, 3607–3619.

Pliotas, C., Dahl, A.C., Rasmussen, T., Mahendran, K.R., Smith, T.K., Marius, P., Gault, J., Banda, T., Rasmussen, A., Miller, S., et al. (2015). The role of lipids in mechanosensation. Nat. Struct. Mol. Biol. *22*, 991–998.

Ranade, S.S., Syeda, R., and Patapoutian, A. (2015). Mechanically activated ion channels. Neuron *87*, 1162–1179.

Rapedius, M., Schmidt, M.R., Sharma, C., Stansfeld, P.J., Sansom, M.S., Baukrowitz, T., and Tucker, S.J. (2012). State-independent intracellular access of quaternary ammonium blockers to the pore of TREK-1. Channels *6*, 473–478.

Renigunta, V., Schlichthorl, G., and Daut, J. (2015). Much more than a leak: structure and function of K(2)p-channels. Pflugers Arch. *467*, 867–894.

Schewe, M., Nematian-Ardestani, E., Sun, E., Musinszki, M., Cordeiro, S., Bucci, G., de Groot, B., Tucker, S.J., Rapedius, M., and Baukrowitz, T. (2016). A non-canonical voltage sensor controls gating in K2P K+ channels. Cell *164*, 937–949.

Sepulveda, F.V., Pablo Cid, L., Teulon, J., and Niemeyer, M.I. (2015). Molecular aspects of structure, gating, and physiology of pH-sensitive background K2P and Kir K+-transport channels. Physiol. Rev. *95*, 179–217.

Stansfeld, P.J., and Sansom, M.S.P. (2011). From coarse grained to atomistic: a serial multiscale approach to membrane protein simulations. J. Chem. Theor. Comput. *7*, 1157–1166.

Sukharev, S., and Sachs, F. (2012). Molecular force transduction by ion channels: diversity and unifying principles. J. Cell Sci. *125*, 3075–3083.

Syeda, R., Florendo, M.N., Cox, C.D., Kefauver, J.M., Santos, J.S., Martinac, B., and Patapoutian, A. (2016). Piezo1 channels are inherently mechanosensitive. Cell Rep. *17*, 1739–1746.

Teng, J., Loukin, S., Anishkin, A., and Kung, C. (2015). The force-from-lipid (FFL) principle of mechanosensitivity, at large and in elements. Pflugers Arch. *467*, 27–37.

Torres-Sanchez, A., Vanegas, J.M., and Arroyo, M. (2015). Examining the mechanical equilibrium of microscopic stresses in molecular simulations. Phys. Rev. Lett. *114*, 258102.

Vanegas, J.M., Torres-Sanchez, A., and Arroyo, M. (2014). Importance of force decomposition for local stress calculations in biomembrane molecular simulations. J. Chem. Theor. Comput. *10*, 691–702.

Vanommeslaeghe, K., Hatcher, E., Acharya, C., Kundu, S., Zhong, S., Shim, J., Darian, E., Guvench, O., Lopes, P., Vorobyov, I., and Mackerell, A.D., Jr. (2010). CHARMM general force field: a force field for drug-like molecules compatible with the CHARMM all-atom additive biological force fields. J. Comput. Chem. *31*, 671–690.

Wu, E.L., Cheng, X., Jo, S., Rui, H., Song, K.C., Davila-Contreras, E.M., Qi, Y., Lee, J., Monje-Galvan, V., Venable, R.M., et al. (2014). CHARMM-GUI membrane builder toward realistic biological membrane simulations. J. Comput. Chem. *35*, 1997–2004.

Yoo, J., and Cui, Q. (2009). Curvature generation and pressure profile modulation in membrane by lysolipids: insights from coarse-grained simulations. Biophys. J. *97*, 2267–2276.

Zhang, X.C., Liu, Z., and Li, J. (2016). From membrane tension to channel gating: a principal energy transfer mechanism for mechanosensitive channels. Protein Sci. *25*, 1954–1964.

Zilberberg, N., Ilan, N., and Goldstein, S.A. (2001). KCNKO: opening and closing the 2-P-domain potassium leak channel entails ‘‘C-type’’ gating of the outer pore. Neuron *32*, 635–648.

STAR★METHODS

KEY RESOURCES TABLE

REAGENT or RESOURCE	SOURCE	IDENTIFIER
Biological Samples		
Xenopus laevis oocytes	Nasco International	http://www.enasco.com
Chemicals, Peptides, and Recombinant Proteins		
1,2-diphytanoyl-sn-glycero-3-phosphocholine	Avanti Polar Lipids	Cat. #850356C
Tetrapentylammonium chloride	Sigma Aldrich	Cat. #258962
Rubidium chloride	Sigma Aldrich	Cat. #215260
Deposited Data		
TREK-2 Down conformation	Dong et al., 2015	PDB: 4XDJ
TREK-2 Up conformation	Dong et al., 2015	PDB: 4BW5
TREK-2 with norfluoxetine bound	Dong et al., 2015	PDB: 4XDK
TWIK-1	Miller and Long, 2012	PDB: 3UKM
TREK-1	http://www.thesgc.org/structures/4twk	PDB: 4TWK
Experimental Models: Organisms/Strains		
Xenopus laevis oocytes	Nasco International	http://www.enasco.com
Recombinant DNA		
Human TREK-2 (*KCNK10*)	Source Bioscience	IRATp970A05115D
Human TREK-1 (*KCNK2*)	Source Bioscience	IRATp970F06122D
Software and Algorithms		
GROMACS 4.6	Hess et al., 2008	http://www.gromacs.org/
MDAnalysis	Michaud-Agrawal et al., 2011	http://www.mdanalysis.org/
UCSF Chimera Dock Prep Tool	Moustakas et al., 2006	https://www.cgl.ucsf.edu/chimera/docs/ContributedSoftware/dockprep/dockprep.html
CGenFF	Vanommeslaeghe et al., 2010	https://cgenff.paramchem.org/
CG2AT	Stansfeld and Sansom, 2011	http://memprotmd.bioch.ox.ac.uk/

CONTACT FOR REAGENT AND RESOURCE SHARING

Further information and requests for resources and reagents should be directed to the lead contact, Stephen Tucker (stephen.tucker@physics.ox.ac.uk).

EXPERIMENTAL MODEL AND SUBJECT DETAILS

Electrophysiological patch clamp recordings used *Xenopus laevis* oocytes obtained from NASCO International (Fort Atkinson, WI 53538 USA). Oocytes were maintained in OR-2 solution (5 mM HEPES, 82.5 mM NaCl, 2.5 mM KCl, 1mM CaCl2, 1 mM $MgCl_2$, 1 mM Na_2HPO_4, pH 7.8) at 17°C prior to injection.

METHOD DETAILS

Computational Methods

The composite 'Down' state structure of TREK-2 was taken from chains A and B of the 4XDJ structure with missing residues atoms modelled using intact chain C and D to create a composite model (Dong et al., 2015). Similar methods were used to obtain the TREK-2 Up state model (PDB 4BW5) used for RMSD comparison and also for the control simulations shown in Figure S1. A membrane-protein system consisting of TREK-2 embedded in a 1-palmitoyl-2-oleoyl-*sn*-glycero-3-phosphocholine (POPC, 16:0, 18:1 PC) bilayer was created by performing a 100 ns protein and bilayer self-assembly using coarse-grained (CG) simulation and MARTINI 2.2 force field (de Jong et al., 2013; Marrink et al., 2007) and then converted to an all-atom (AT) system using CG2AT (Stansfeld and Sansom, 2011). The final all-atom system contained the protein with 240 lipids, 25,000 TIP3P waters and 150 mM KCl, and was simulated using the Charmm36 force field (Best et al., 2012). For distances compared to TREK-1 the PDB 4TWK structure

was used. Membrane thickness was calculated as the distance between the headgroup phosphates. Lateral pressure profiles were calculated from CG simulations of symmetrical bilayers in either the absence or presence of embedded TREK-2 adapting recent methods (Ollila et al., 2009; Torres-Sanchez et al., 2015; Vanegas et al., 2014). Briefly, this computes the local stress tensor in 3D based on the Hardy stress ($\boldsymbol{\sigma}$) definition using the Irving-Kirkwood-Noll (IKN) procedure. The lateral pressure (P_L) was obtained using the following relations and the pressure profile calculated with 0.1 nm grid size and averaged over 4,000 frames (i.e. 800 ns):

$P_L(z) = (P_{\|}(z) - P_{\perp}(z))$, $\boldsymbol{P} = -\boldsymbol{\sigma}$, and $P_{\|}(z) = (P_{xx}(z)+P_{yy}(z))/2$

To investigate the stretch-induced changes in TREK-2, NPT ensemble simulations of the ‘Down’ state conformation were performed; the bilayer plane (xy-plane) pressure was varied between -50, -30, -20, -5 and 1 bar, whereas the pressure in bilayer normal (z) direction kept at +1 bar and the temperature at 310 K. RMSD analysis against the Down and Up states were used to determine whether structural transitions occurred. TREK-2 was also simulated in 1,2-dilauroyl-*sn*-glycero-3-phosphocholine (DLPC, 12:0 PC) at normal pressure. For TREK-2 in DLPC, the bilayers were set up using the CHARMM-GUI (Brooks et al., 2009; Wu et al., 2014), and performed at 323 K to maintain bilayer fluidity. Simulations of the non-mechanosensitive TWIK-1 in POPC were similar to those described previously (Aryal et al., 2014, 2015a) using the CHARMM-36 force field.

For simulations with NFx, ligands were placed in their binding sites on the TREK-2 Down state by alignment with the TREK-2/NFx crystal structure (4XDK). Hydrogens were added using USCF chimera Dock Prep tool (Moustakas et al., 2006) and drug topology generated using CGenFF (Vanommeslaeghe et al., 2010). To equilibrate NFx, a 2 ns simulation was conducted with Cα atom positional restraints before unrestrained simulations were then performed. As a control, stretch simulation at -50 bar was repeated with both NFx ligands deleted at the start of the stimulation. Similarly, for the lipid-deletion simulation, the POPC molecules bound between TM2/TM4 was deleted at the end of a 100 ns of stretch simulation, and the stretch protocol then restarted for an additional 100 ns (Faraldo-Gomez et al., 2002).

All simulations were done using GROMACS 4.6 (Hess et al., 2008) with a 2 fs integration time step. The total all-atom simulation time for the 26 simulations in this study was > 6 μs. Each simulation ra for at least 200 ns. A periodic boundary condition was applied in all directions. For all-atom simulations, the temperature and semi-isotropic pressure with a compressibility of 4.5×10^{-5} bar^{-1} were controlled by a velocity-rescale coupling algorithm and Parrinello-Rahman barostat (Bussi et al., 2007). Electrostatic interactions were calculated using the particle mesh Ewald (PME) with 0.12 nm Fourier spacing, and LINCS constraints applied to all bonds (Hess et al., 1997). All repeat simulations were performed with differences in initial configuration or velocity. For CG simulations, the integration time step was 20 fs, the temperature and semi-isotropic pressure were set to 310 K and 1 bar in both directions with compressibility of 5×10^{-6}/bar controlled by Berendsen coupling (Berendsen et al., 1984). Electrostatics and van der Waals interactions were treated as shifts applied between 0-1.2 nm, and 0.9-1.2 nm, respectively. VMD was used for visualization and RMSD calculations (Humphrey et al., 1996). Analysis was done using GROMACS and MDanalysis (Michaud-Agrawal et al., 2011). Cross-sectional areas were obtained using CHARMM-GUI.

Electrophysiological Methods

For expression of channels in *Xenopus* oocytes the pipette solution contained (in mM): 120 KCl, 10 HEPES and 3.6 $CaCl_2$ (pH 7.4 adjusted with KOH/HCl). Bath solutions contained (in mM): 120 KCl or RbCl, 10 HEPES, 2 EGTA, 1 Pyrophosphate. Bath solutions were applied via a multi-barrel pipette system to the cytoplasmic side of giant excised membrane patches. Human TREK-1 currents were recorded at room temperature at +60 mV. Data were sampled at 10 kHz and filtered at 3 kHz. Planar lipid bilayer recordings of ‘crystallization grade’ purified truncated human TREK-2 protein (residues 67-340), i.e. identical to the protein used to obtain the TREK-2 structures, were performed using a Nanion Port-a-Patch recording system and Vesicle Prep Pro for reconstitution of proteins into giant unilamellar vesicles made from DPhPC. Data were sampled at 250 kHz and filtered at 10 kHz. Negative pressure (-80 mmHg) was applied to the bilayer using the Nanion Port-a-Patch suction control unit.

QUANTIFICATION AND STATISTICAL ANALYSIS

Electrophysiology

All quoted error values represent the standard error of the mean with n representing the relevant number of independent experiments.

DATA AND SOFTWARE AVAILABILITY

All simulation trajectories are available from the lead contact upon request. All software described in the Method Details is available from the sites listed in the table.

Structure, Volume *25*

Supplemental Information

Bilayer-Mediated Structural Transitions Control Mechanosensitivity of the TREK-2 K2P Channel

Prafulla Aryal, Viwan Jarerattanachat, Michael V. Clausen, Marcus Schewe, Conor McClenaghan, Liam Argent, Linus J. Conrad, Yin Y. Dong, Ashley C.W. Pike, Elisabeth P. Carpenter, Thomas Baukrowitz, Mark S.P. Sansom, and Stephen J. Tucker

Supplementary Information

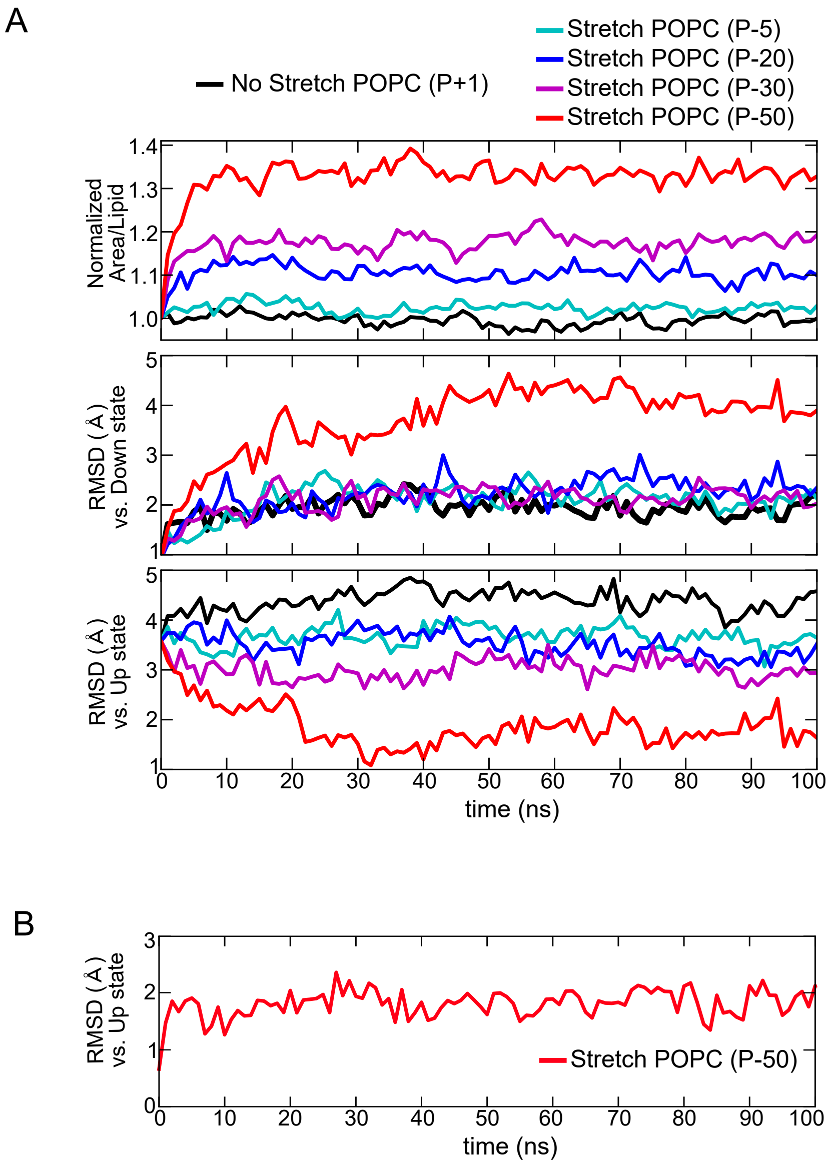

Figure S1. Related to Figure 1.

Development of stretch protocol. (**A**) Top: Increase in bilayer surface area per lipid in response to application of increasing levels of membrane stretch. Bilayer surface tensions have been estimated by using $\gamma = Z(P_{\perp} - P_{\parallel})$, where Z is average box size in bilayer normal direction, $P_{\perp}$ and $P_{\parallel}$ is pressure in bilayer normal and bilayer plane, respectively. The estimated average bilayer tensions correlating with the pressure

protocols described in this figure (P-5, P-20, P-30, and P-50 bar) are: 7.6, 24.6, 34.3, and 50.3 mN m^{-1}, respectively. Middle: Cα RMSD for M1-M4 of TREK-2 against the Down state crystal structure during 200 ns of simulation for different levels of membrane stretch in a POPC bilayer. At P-50 (-50 bar) there is rapid movement towards the Up state within 50 ns, but not in the absence of stretch (black). Bottom: Similar analysis of RMSD against the Up state crystal structure showing movement towards the Up state at different membrane tensions. (**B**) Simulation of the Up state structure (4BW5) as the starting conformation shows that this conformation is extremely stable in response to the P-50 stretch protocol and exhibits little or no movement of the TM-helices (RMSD 1-2 Å).

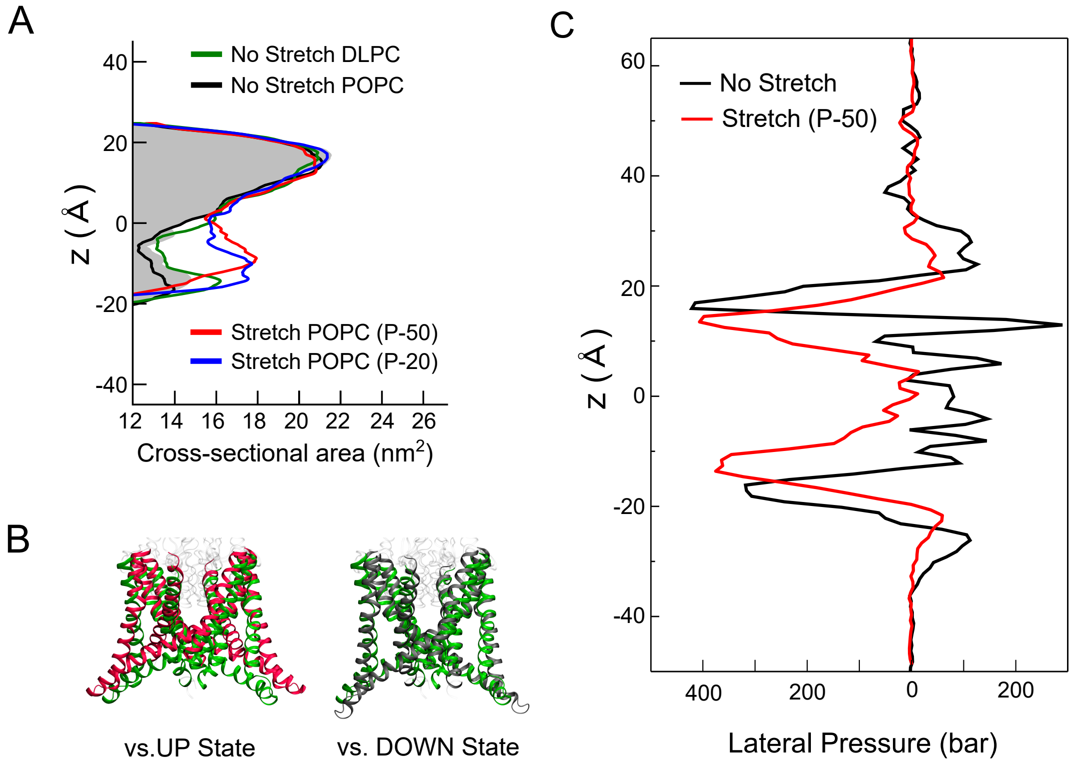

Figure S2. Related to Figure 2.

Effect of changes in the pressure profile and bilayer thickness. (**A**) Change in cross-sectional area of TREK-2 Down state during simulation in the different bilayers used in this study. Stretch at -50 bar (red) and also -20 bar (blue) produce similar increases in cross-sectional area but over different timescales. By marked contrast, the thinner DLPC bilayer (i.e. hydrophobic mismatch) is not sufficient to drive movement towards the Up state (green). Grey shaded area represents the Down state crystal structure (4XDJ). (**B**) This lack of movement is also illustrated by comparison of the TREK-2 structure after simulation in a DLPC bilayer (green) with the Up (red) and Down state (dark grey) crystal structures. (**C**) Pressure profiles calculated for stretched (red) and unstretched (black) POPC bilayers with TREK-2 protein embedded within the bilayer. These profiles are more complex but membrane stretch still reduces the positive pressures within the core of the bilayer and changes the tension at the interfacial regions.

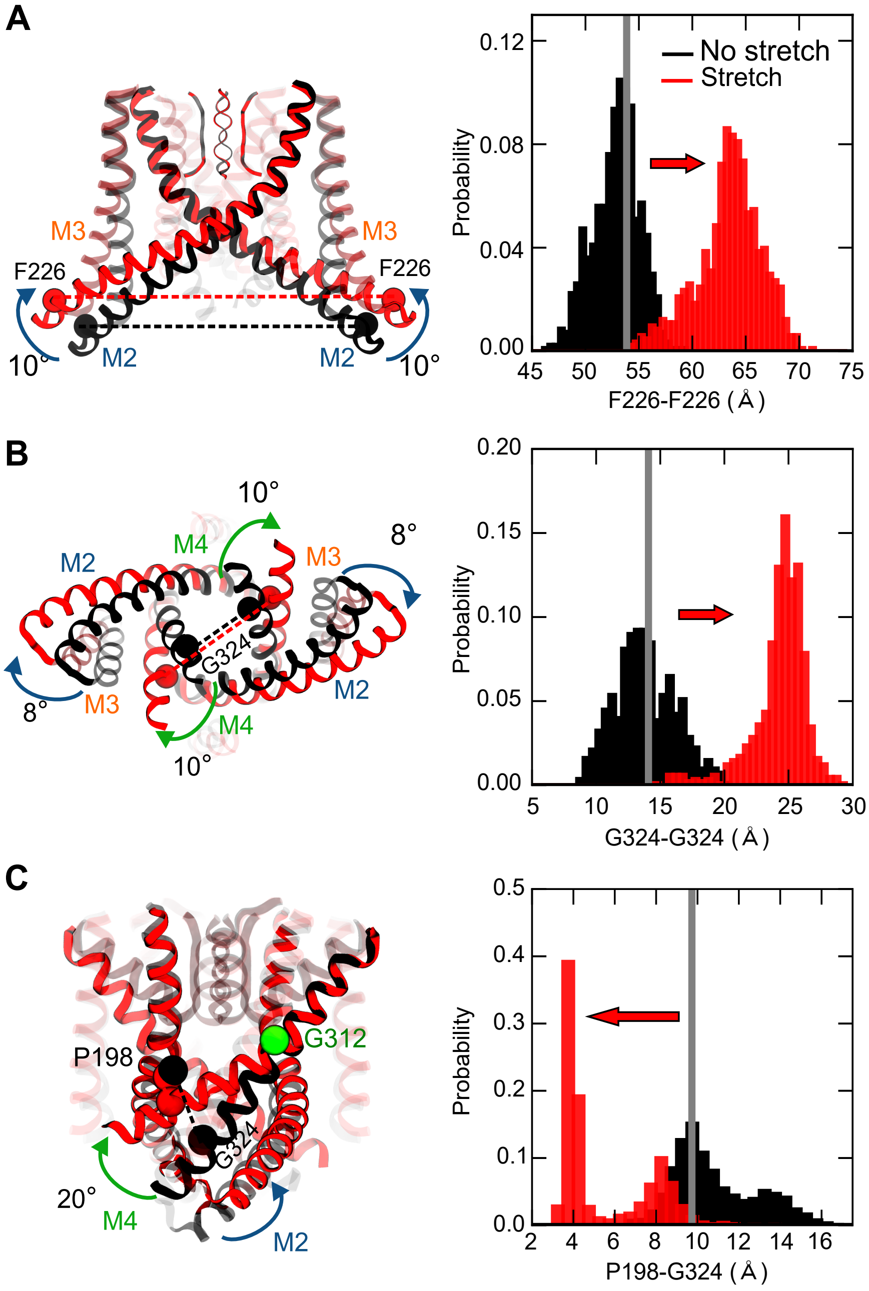

Figure S3. Related to Figure 3.

Stretch induced changes in the structure of TREK-2. (**A**) Left: Stretch increases the tilt angle of the bow-like M2 helices by 10° and also increases the distance between them (measured between F226 residues). Right: Distribution of distances between F226

during stretched (red) and unstretched simulations (black) showing >10 Å increase upon stretched. Vertical grey line indicates distance in the Down state crystal structure. Histograms represent the distribution of distances between the specified residues (sampled every 1 ns) during the stretched and unstretched simulations. (**B**) Rotated view up into channel pore revealing additional 8° twist in M2 (blue arrow) and 10° twist in M4 (green arrow). Stretch increases the distance between G324 residues on M4 as also shown in the distributions. (**C**) Side view showing the 20° kink in M4 upon membrane stretch. M4 kinks at the conserved glycine hinge (G312; green dot) and subsequent movement towards M2 closes and seals the fenestration. Distance distributions show closure of fenestration upon membrane stretch (measured between G324 on M4 and P198 on M2 of the adjacent chain).

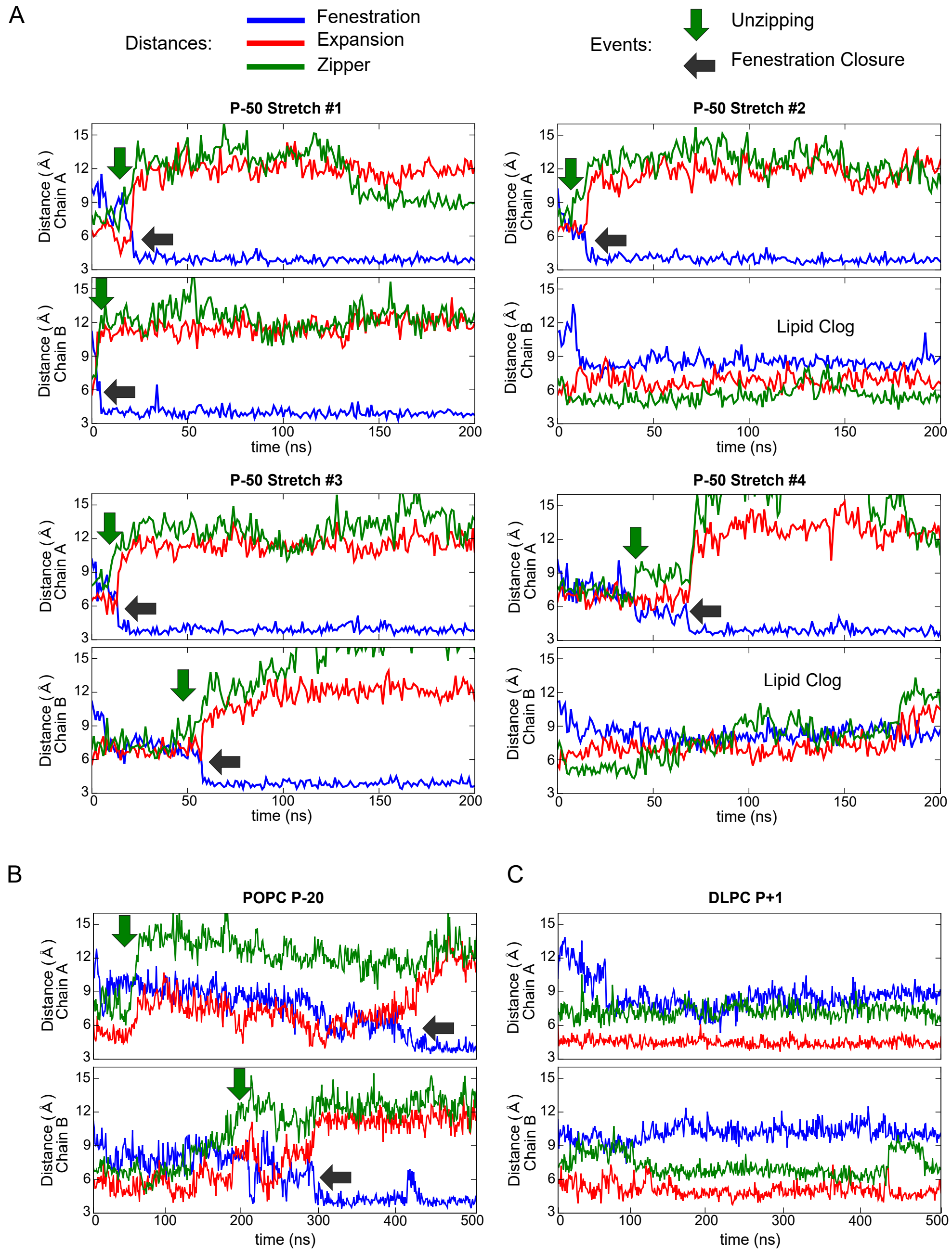

Figure S4. Related to Figure 3.

Sequential and stepwise rearrangement of the TM helices in response to membrane stretch. (**A**) Similar to the data shown in Figure 3B, changes in the fenestration (blue), expansion (red) and zipper (green) distances over time for the 4 stretch simulations at -50 bar in a POPC bilayer. Profiles for both chains A and B within each TREK-2 channel are shown. Green arrows indicate the 'unzipping' of the

interactions between the lower sections of M4 and M3. The black arrows indicate full closure of the fenestration. Note in chain B of repeats #2 and #4 there is no stepwise transition or closure of the fenestration due to presence of a lipid within the fenestration (lipid clog). (**B**) A similar sequence of structural transitions occurs when the POPC bilayer is stretched at a lower pressure of -20 bar. (**C**) A thinner DLPC bilayer fails to drive these structural transitions suggesting that hydrophobic mismatch alone is not sufficient.

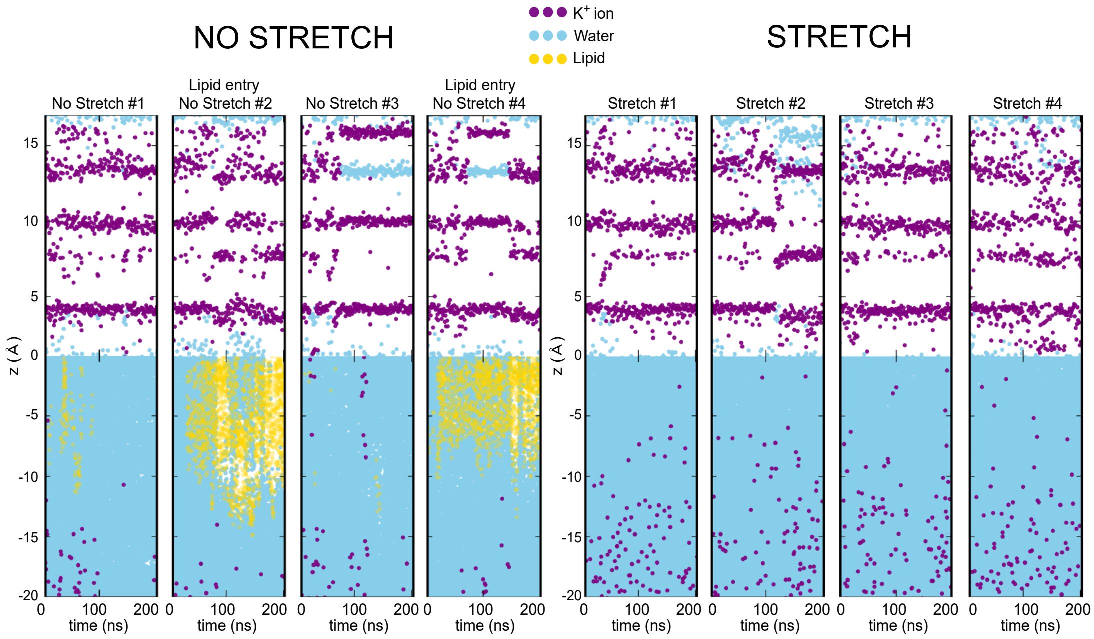

Figure S5. Related to Figure 6.

Effects of membrane stretch on the selectivity filter and hydration of the inner pore cavity. Left: Occupancy along the axis of the pore and inner cavity for ions (purple), water (cyan) and lipid tails (yellow) shown against time of the four independent 200 ns simulations performed in the absence of membrane stretch. Transient dewetting occurs and correlates with the presence of lipid tails (yellow dots) penetrating the cavity, but only in two of the four simulations. Right: As in left panel, but in the presence of membrane stretch (-50 bar). Note the cavity remains fully hydrated in all four simulations (see Figure 6).
